# Comparison of SNP-based subtyping workflows for bacterial isolates using WGS data, applied to *Salmonella enterica* serotype Typhimurium and serotype 1,4,[5],12:i:-

**DOI:** 10.1371/journal.pone.0192504

**Published:** 2018-02-06

**Authors:** Assia Saltykova, Véronique Wuyts, Wesley Mattheus, Sophie Bertrand, Nancy H. C. Roosens, Kathleen Marchal, Sigrid C. J. De Keersmaecker

**Affiliations:** 1 Platform Biotechnology and Molecular Biology, Scientific Institute of Public Health, Brussels, Belgium; 2 Department of Information Technology, IDLab, Ghent University, IMEC, Ghent, Belgium; 3 Bacterial Diseases Division, Communicable and Infectious Diseases, Scientific Institute of Public Health, Brussels, Belgium; 4 Department of Plant Biotechnology and Bioinformatics, Ghent University, VIB, Ghent, Belgium; 5 University of Pretoria, Pretoria, South Africa; Laboratoire National de Santé, LUXEMBOURG

## Abstract

Whole genome sequencing represents a promising new technology for subtyping of bacterial pathogens. Besides the technological advances which have pushed the approach forward, the last years have been marked by considerable evolution of the whole genome sequencing data analysis methods. Prior to application of the technology as a routine epidemiological typing tool, however, reliable and efficient data analysis strategies need to be identified among the wide variety of the emerged methodologies. In this work, we have compared three existing SNP-based subtyping workflows using a benchmark dataset of 32 *Salmonella enterica* subsp. *enterica* serovar Typhimurium and serovar 1,4,[5],12:i:- isolates including five isolates from a confirmed outbreak and three isolates obtained from the same patient at different time points. The analysis was carried out using the original (high-coverage) and a down-sampled (low-coverage) datasets and two different reference genomes. All three tested workflows, namely CSI Phylogeny-based workflow, CFSAN-based workflow and PHEnix-based workflow, were able to correctly group the confirmed outbreak isolates and isolates from the same patient with all combinations of reference genomes and datasets. However, the workflows differed strongly with respect to the SNP distances between isolates and sensitivity towards sequencing coverage, which could be linked to the specific data analysis strategies used therein. To demonstrate the effect of particular data analysis steps, several modifications of the existing workflows were also tested. This allowed us to propose data analysis schemes most suitable for routine SNP-based subtyping applied to *S*. Typhimurium and *S*. 1,4,[5],12:i:-. Results presented in this study illustrate the importance of using correct data analysis strategies and to define benchmark and fine-tune parameters applied within routine data analysis pipelines to obtain optimal results.

## Introduction

Efficient surveillance and outbreak detection are important to limit economic and health burden of infectious diseases. A key component of infection control and prevention tasks performed by the public health authorities worldwide is subtyping of microorganisms. Discrimination and detailed characterisation of isolates belonging to the same species is necessary for detection and investigation of outbreaks, including tracing of infection transmission events, finding infection sources and describing the epidemiology behind the outbreaks. Moreover, classification of isolates at sub-species level allows monitoring composition and dynamics of circulating microbial populations which is necessary, for example, for early detection of emerging pathogen subtypes and evaluation of the intended effects of public health measures such as vaccination.

During the last decade, there has been a continuous evolution of bacterial subtyping techniques, moving from phenotypic methods such as serotype, phage-type and antibiogram towards more informative, standardized and therefore portable molecular and genotypic methods such as pulsed-field gel electrophoresis (PFGE), multiple-locus variable-number of tandem repeats analysis (MLVA) and multilocus sequence typing (MLST) [1]. Despite this improvement, currently used subtyping technologies show multiple drawbacks, most important being the fact that their discriminatory potential is in many cases not sufficient to differentiate between closely related strains, compromising our ability to unambiguously detect outbreaks and perform epidemiological investigation. The ongoing decrease of the cost and turnaround time of the next-generation sequencing (NGS) technologies has made whole genome sequencing (WGS) of isolates a viable option for replacing the current subtyping technologies. Whole genome sequencing not only offers the highest level of resolution to discriminate between isolates, but also allows extracting additional useful information e.g. for prediction of antibiotic resistance [2–4] and serotype [5,6], and detection of virulence factors [7,8]. This technology has already been applied for outbreak investigations and epidemiological studies of important human pathogens [9–13] and is increasingly being used to subtype some pathogens routinely in Europe [14].

One of the factors slowing down the wide routine implementation of WGS for daily surveillance by public health institutes is the data analysis: extracting typing information from the large number of short reads produced by the currently most used sequencing platforms is non-trivial [15]. While the data acquisition protocol is relatively comparable for different microorganisms, no single data analysis methodology suits all bacterial species given their different genomic diversity and population structures. The core genome MLST (cgMLST)- and the single nucleotide polymorphism (SNP)-based approaches are the two most commonly used principles for the retrieval of subtyping information from WGS data [16]. The cgMLST assigns a sequence type based on the allele calls observed for each of >1000 of core genes predefined in an organism-specific typing scheme. The cgMLST has shown to be very useful in multiple typing efforts, it allows the global exchange of data and is easy to standardize but depends on the availability of generally accepted and sustained typing schemes that are accessible through international databases for each individual pathogenic microorganism [17]. Currently cgMLST schemes are being developed and maintained for important human pathogens [18–21], but such efforts will likely bypass the less significant species and subspecies. Typing approaches based on SNPs on the other hand are more flexible as they do not require a predefined scheme. Rather they infer phylogenies using all SNPs observed between the genomes of the analysed isolates and a reference genome. SNP-based approaches provide an exceptionally high subtyping resolution [19,22,23], but tend to be computationally demanding. Therefore, at least currently, SNP-based subtyping is less suitable for characterisation of a large number of distantly related isolates. It is more often being applied to distinguish between isolates that have been identified as closely related using subtyping methods such as k-mer and MLST [24,25], or even cgMLST [16].

Typing results obtained by SNP-based identification tend to be less standardized as the results largely depend on the used data processing strategies and parameter settings of the applied workflows. For many human pathogens such as *Salmonella enterica* subsp. *enterica* more than one SNP-based data analysis workflow have been described, some of them currently being applied or tested for the use in routine settings in different larger public health research centres and institutions [10,26–33]. As these approaches differ from each other in the underlying strategies and tools, they are very likely producing an at least slightly different output. The extent of such differences, as well as their practical implications for some important end-users of the pipelines, namely the public health institutes, reference laboratories and centres and hospitals have not been sufficiently investigated. Besides for public health, consequences of actions taken based on WGS-based subtyping can have large economic and public image implications for industry.

In this study we aimed at comparing data analysis tools for WGS-based typing of bacterial isolates using the SNP-based principle. We tested three existing SNP-based typing methodologies that have been used for subtyping of *S*. *enterica* subsp. *enterica* and compared their performance using sequencing data from 32 *S*. *enterica* subsp. *enterica* serovar Typhimurium (*S*. Typhimurium) and serovar 1,4,[5],12:i:- (*S*. 1,4,[5],12:i:-) isolates. S. Typhimurium belongs to the most common foodborne pathogens worldwide [34], often causing outbreaks associated with consumption of eggs, pork, beef and poultry meat [35]. In recent years, the added value of WGS for epidemiological investigation of *Salmonella* has been demonstrated [10,36–39]. For the benchmark dataset, epidemiological information was available that was used to validate the results. We compared the epidemiologic concordance and typing resolution of the different workflows, and evaluated how these factors were affected by the coverage of the sequencing data, and the choice of the reference genome. The observed differences were associated with the underlying workflow architecture and used parameters. Taking into account the implication of these differences for routine subtyping in a public health institution, guidelines for optimal workflow implementation were designed and workflows with most suitable performance characteristics for routine SNP-based subtyping with S. Typhimurium and *S*. 1,4,[5],12:i:- were identified.

## Materials and methods

### Isolates

32 isolates of *S*. Typhimurium and the *S*. Typhimurium-like *S*. 1,4,[5],12:i:- [40] were selected from the collections of 2011, 2012 and 2013 of the Belgian National Reference Centre for *Salmonella* and *Shigella* (NRCSS) (Table 1). The selection included the following groups of different epidemiological origin: (1) five *S*. 1,4,[5],12:i:- isolates involved in the same outbreak in a day nursery in 2011, and previously characterized as phage type DT138 and MLVA profile 3-13-11-NA-211, together with two *S*. Typhimurium out-group isolates sampled during the same period as the outbreak; (2) seven *S*. 1,4,[5],12:i:- and *S*. Typhimurium isolates of identical phage type, and with an identical or a frequently occurring MOL-PCR profile as observed for the outbreak and out-group isolates; (3) three *S*. Typhimurium isolates obtained from the same patient at different time points (not related to the outbreak, sampled over a period of about 1.5 months); (4) 15 background *S*. 1,4,[5],12:i:- and *S*. Typhimurium isolates with frequently occurring combinations of MOL-PCR profiles (15-1-1 and 15-3-1), phage types (DT193 and DT120) and MLVA profiles (3-12-10-NA-211 and 3-13-11-NA-211) [41]. Phage type and MLVA data were provided by the NRCSS and were collected as described by Wuyts et al. [42]. Multiplex Oligonucleotide Ligation-PCR (MOL-PCR) data has been determined previously by Wuyts et al. [41]. Sequence type was determined from the WGS data using MLST-1.8 Server from Centre of Genomic Epidemiology [43].

**Table 1:** Overview and classical typing data of *S*. Typhimurium and *S*. 1,4,[5],12:i:- isolates in the study.

ID	Serovar	Isolation date	MOL-PCR profile	Phage type	MLVA profile	ST	Additional information
11–0596	1,4,[5],12:i:-	15/01/2011	15-1-1	DT138	3-13-11-NA-211	ST-34	Outbreak
11–1163	1,4,[5],12:i:-	28/03/2011	15-1-1	DT138	3-13-11-NA-211	ST-34	Outbreak
11–1164	1,4,[5],12:i:-	28/03/2011	15-1-1	DT138	3-13-11-NA-211	ST-34	Outbreak
11–1165	1,4,[5],12:i:-	28/03/2011	15-1-1	DT138	3-13-11-NA-211	ST-34	Outbreak
11–1166	1,4,[5],12:i:-	26/03/2011	15-1-1	DT138	3-13-11-NA-211	ST-34	Outbreak
11–0600	Typhimurium	04/02/2011	15-3-1	RDNC	3-14-11-NA-211	ST-34	Out-group outbreak
11–1160	Typhimurium	10/04/2011	1.21×10^18^−2.58×10^11^−4199	DT104	3-14-18-14-311	ST-19	Out-group outbreak
S13BD00332	Typhimurium	02/03/2013	1.50×10^8^−1.69×10^11^−1155	ND	3-14-14-5-311	ST-19	One patient
S13BD00591	Typhimurium	25/03/2013	1.50×10^8^−1.69×10^11^−1155	ND	3-14-14-5-311	ST-19	One patient
S13BD00844	Typhimurium	19/04/2013	1.50×10^8^−1.69×10^11^−1155	ND	3-14-14-5-311	ST-19	One patient
12–2003	1,4,[5],12:i:-	29/06/2012	15-1-1	DT120	3-12-10-NA-211	ST-34	Background**
12–2203	1,4,[5],12:i:-	11/07/2012	15-1-1	DT120	3-12-10-NA-211	ST-34	Background**
12–2460	1,4,[5],12:i:-	22/06/2012	15-1-1	DT120	3-12-10-NA-211	ST-34	Background**
12–2455	1,4,[5],12:i:-	26/07/2012	15-1-1	DT193	3-12-10-NA-211	ST-34	Background**
12–2599	1,4,[5],12:i:-	07/08/2012	15-1-1	DT193	3-12-10-NA-211	ST-34	Background**
12–2730	1,4,[5],12:i:-	14/08/2012	15-1-1	DT193	3-12-10-NA-211	ST-34	Background**
12–1558	1,4,[5],12:i:-	22/05/2012	15-1-1	DT193	3-13-11-NA-211	ST-34	Background**
12–2314	1,4,[5],12:i:-	17/07/2012	15-1-1	DT193	3-13-11-NA-211	ST-34	Background**
12–2379	1,4,[5],12:i:-	29/07/2012	15-1-1	DT193	3-13-11-NA-211	ST-34	Background**
12–3792	Typhimurium	08/10/2012	15-3-1	DT120	3-12-10-NA-211	ST-34	Background**
12–3907	Typhimurium	14/10/2012	15-3-1	DT120	3-12-10-NA-211	ST-34	Background**
12–3990	Typhimurium	23/10/2012	15-3-1	DT120	3-12-10-NA-211	ST-34	Background**
12–0084	Typhimurium	13/01/2012	15-3-1	DT193	3-12-10-NA-211	ST-34	Background**
12–0161	Typhimurium	21/01/2012	15-3-1	DT193	3-12-10-NA-211	ST-34	Background**
12–3663	Typhimurium	30/09/2012	15-3-1	DT193	3-12-10-NA-211	ST-34	Background**
12–3558	1,4,[5],12:i:-	25/09/2012	15-1-1	DT138	3-12-11-NA-211	ST-34	Background*
12–3582	1,4,[5],12:i:-	11/09/2012	15-1-1	DT138	3-12-11-NA-211	ST-34	Background*
12–3583	1,4,[5],12:i:-	11/09/2012	15-1-1	DT138	3-12-11-NA-211	ST-34	Background*
12–2984	1,4,[5],12:i:-	27/08/2012	15-1-1	RDNC	3-12-11-NA-211	ST-34	Background*
12–2998	1,4,[5],12:i:-	Not known	15-1-1	RDNC	3-12-11-NA-211	ST-34	Background*
12–3067	1,4,[5],12:i:-	04/09/2012	15-1-1	RDNC	3-12-11-NA-211	ST-34	Background*
12–3444	Typhimurium	22/09/2012	1.21×10^18^−2.58×10^11^−4199	DT104	3-16-16-13-311	ST-19	Background*

Background: background isolates; MLVA: multiple-locus variable-number of tandem repeats analysis; MOL-PCR: multiplex oligonucleotide ligation-PCR; NA: absence of a PCR amplicon in MLVA; ND: not determined as phage type was replaced by MLVA at the NRCSS; One patient: isolates obtained from one patient at different time points; Outbreak: isolates related to an epidemiologically confirmed outbreak; Out-group outbreak: out-group isolates that were collected during the same period as the outbreak and that were used in the original outbreak investigation; RDNC: reacts-but-does not confirm, used for isolates that give lysis reactions with the bacteriophages, but these reactions do not match any of the patterns that define a certain phage type [44]; ST: sequence type

(*) background isolates of identical phage type, and with an identical or a frequently occurring MOL-PCR profile as observed for the outbreak and out-group isolates

(**) background isolates with frequently occurring combinations of MOL-PCR profiles, phage types and MLVA profiles.

### Genomic DNA purification and sequencing data acquisition

The isolates were grown overnight in brain-heart infusion (BHI) broth, and genomic DNA was obtained from a single colony using Qiagen Genomic-tip 100/G kit according to the manufacturer’s instructions. The sequencing was carried out at EMBL GeneCore facility on an Illumina HiSeq 2000, obtaining 100 bp paired-end reads. Forty isolates were multiplexed on a single lane. The raw reads used in this study have been deposited at the WIV-ISP—*Salmonella*, Oct 03 '17 BioProject at NCBI (PRJNA412988). Sequencing quality was uniformly high, with mean PHRED scores laying above 34 for all samples.

### Quality control of sequencing data

Prior to running the workflows, the quality of the sequencing data was analysed. First, general statistics were collected using FastQC 0.11.4, a widely-used quality control application for data generated on Illumina platform [45]. Second, we analysed the sequencing dataset using Qualimap 2.2.1, a tool that produces descriptive statistics on mapped NGS data, allowing to detect sequencing and/or mapping biases between the samples [46]. Therefore, raw reads were mapped on the LT2 (NC_003197.1) and the SL1344 (NC_016810.1) reference genomes using *mem* function of BWA 0.7.12 [47], upon which the produced bam files were analysed using *multi-sample BAM QC* function of Qualimap applied at default parameters. This analysis indicated that for isolates 11–0596, 12–1558, 12–2203, 12–3444, 12–3583, 12–3990, 12–2998, S13BD00332, S13BD00844 and 12–3558, the fractions of genomic positions covered above a given number of reads was lower compared to the values observed for isolates showing the same average coverage (S1 and S2 Figs). This was not associated with the average coverage or duplication rate (S1 Fig), and was likely caused by an uneven sequencing depth across the genome (S2 Fig). Despite this issue, the fraction of any of the two reference genomes covered with more than 10 reads exceeded 85% in all samples and therefore we considered data quality to be acceptable for subtyping.

### SNP-based typing

**Fig 1:**
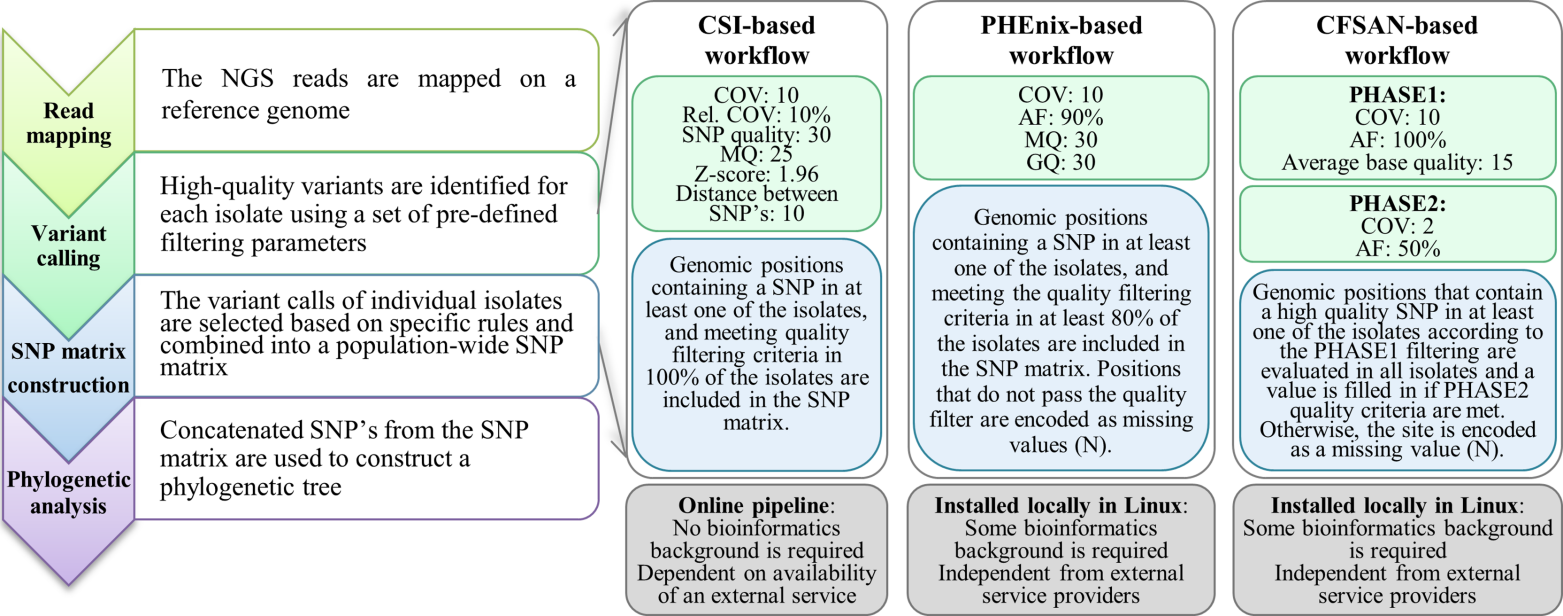
Schematic representation of the tested SNP-based subtyping workflows. Left: data analysis steps of a SNP-based subtyping workflow; Right: details of variant calling and SNP matrix construction steps of the tested SNP-based subtyping workflows. AF: allele frequency, COV: coverage, GQ: genotype quality, MQ: mapping quality, Rel. COV: relative coverage.

For each workflow the following main steps can be distinguished (1) read mapping: alignment of the reads against a reference genome; (2) variant calling: identification of high-quality SNP positions for each isolate; (3) SNP matrix construction: building of a dataset-wide SNP matrix containing reliable genomic polymorphic sites; and (4) phylogenetic analysis: generation of a phylogenetic tree from the concatenated SNP matrix positions (Fig 1). As differences between workflows in the specificities of each of the steps are likely to affect typing results, when applicable, we have performed additional tests to associate observed differences in performance to the specificities of the workflow implementations, hereby focusing on the variant calling and SNP matrix construction. While the read mapping is also highly influential for the typing outcome, we believe that the effect of the read mapping step is difficult to evaluate separately from the variant selection step as the parameters used during variant selection in a particular workflow are typically designed taking into account the specificities of the read mapping software.

#### CSI Phylogeny-based subtyping workflow

CSI Phylogeny-based subtyping (further referred to as the CSI-based workflow) was performed using CSI Phylogeny-1.2 (https://cge.cbs.dtu.dk/services/CSIPhylogeny-1.2, [26]), an online service for inferring phylogeny based on WGS data, that is made available by the Centre of Genomic Epidemiology of the Technical University of Denmark (DTU). After completion of the analysis, a newer version has become available (1.4), but the differences in the final output between the versions are neglectable (data not shown). The pipeline was run with default parameters. The raw reads were uploaded, and the analysis was submitted with following parameters: a minimal depth at SNP positions of 10 reads, a minimal relative depth at SNP positions of 10%, a minimal distance between SNPs of 10 bp, a minimal Z-score of 1.96, a minimal SNP quality of 30 and a minimal read mapping quality of 25. The Z-score expresses the confidence with which a base was called at a given position, with a Z-score of 1.96 corresponding to a p-value of 0.05. The minimal SNP quality and the minimal read mapping quality are PHRED-based, and can be converted to probabilities to respectively observe an incorrect SNP call or a wrong read alignment using the general formula. The pipeline is described in Kaas et al. [26], and consists briefly of the following steps: reads are mapped on the reference genome with BWA mem, and SNPs are called with the *mpileup* tool from SAMTools [48]. The SNPs are filtered according to the submitted parameters to retain high-quality instances (to this end, read depth is calculated with the genomeCoverageBed tool from BEDTools [49], read mapping quality is obtained from BWA mem, and SNP quality is calculated with SAMTools). Next, a SNP matrix is constructed by evaluating all positions where a SNP was called in at least one of the isolates, and retaining positions that meet the minimal depth check and the Z-score requirements in all isolates. Positions that fail validation in at least one of the mappings are excluded from the SNP matrix. Retained positions are concatenated per isolate to create a multiple FASTA file that is used for phylogenetic analysis.

#### PHEnix-pipeline based subtyping workflow

The PHEnix-pipeline for subtyping version 1.2, made available by Public Health England (PHE) was obtained from https://github.com/phe-bioinformatics/PHEnix. The analysis (further referred to as the PHEnix-based workflow) was performed as described by Ashton et al. [10]. Reads were quality-trimmed using Trimomatic 0.3 [50] and mapped to the reference with BWA 0.7.12 *mem* at default parameters. Duplicate reads were marked with *MarkDuplicates* function of Picard-tools 2.0.1 [51]. The reads were realigned with the *IndelRealigner* function of the GenomeAnanlysisTK (GATK) 3.4–46 toolbox [52] and BAM files were provided to the PHEnix-pipeline, where variant calling was performed with *UnifiedGenotyper* tool from GATK, with a standard variant calling confidence and a standard variant emitting confidence of 30 and the ploidy of the samples set to 1. Raw SNPs were filtered, retaining high-quality variants showing a minimal mapping quality and a minimal genotype quality of 30, minimal read depth of 10 and a minimal allele frequency of 90%. Genomic positions that did not satisfy these requirements were masked as unknown state characters (N) in the consensus sequence. Next, a SNP matrix was constructed using positions for which a SNP was found in at least one isolate, and that could be called in at least 80% of the isolates. The selected positions were extracted and concatenated per isolate in a multiple FASTA file for phylogenetic analysis.

#### Adapted PHEnix-based workflow using an alternative SNP matrix construction strategy

In all of the tested workflows, the positions that are missing from the SNP matrix for some of the isolates (e.g. because of an insufficient coverage of the reference genome or because the region is absent from the genome of the tested isolate), are replaced by an unknown state character (N). Because of the ease of implementation, another strategy is sometimes applied where the missing values are replaced by the reference values [53–56]. In order to demonstrate the effect of this strategy, we have constructed a custom SNP-based subtyping workflow, further called adapted PHEnix-based workflow, in which the variant selection procedure of the PHEnix-based workflow was combined with the SNP matrix construction step where genomic positions that did not meet quality requirements were considered equal to reference values rather than being masked. All positions with a high-quality SNP in at least one of the isolates were concatenated and the produced multiple FASTA file was used for phylogenetic tree construction.

#### CFSAN SNP Pipeline-based subtyping workflow

CFSAN SNP Pipeline-based subtyping (further called the CFSAN-based workflow) was performed using a locally installed CFSAN pipeline 0.6.1 [28], that has been released by the U.S. Food and Drug Administration (FDA). The pipeline was run with the parameters applied by Wilson et al. [29] for subtyping of *S*. *enterica* serotype Tennessee. Raw reads were provided as input of the pipeline, where they were mapped to the reference with Bowtie 2.2.4 aligner at default parameters [57]. Read mapping statistics were gathered with *mpileup* function of SAMTools, using a minimal read mapping quality of 0 and a minimal base quality of 13. Next, a two-phase variant selection procedure was applied where in the first phase SNPs were called with *mpileup2snp* tool from VarScan 2.3.9, with a minimal average base quality of 15, a minimal read depth of 10 and a minimal allele frequency of 100%. Positions with a SNP that fulfilled all these criteria in at least one of the isolates were combined to a list. In the second phase, nucleotide states at the listed positions were re-determined for all isolates, including those isolates for which the more stringent criteria from the first phase were not fulfilled, using more relaxed rules: a) if there were less than 2 reads mapped at a position, it was coded with the unknown state character (N); b) if different nucleotides were observed at the position, the nucleotide with frequency larger than 50% was the consensus call for that position; and c) in case that none of the called nucleotides had a frequency larger than 50%, the position was coded with the unknown state character (N) for that particular isolate. The two-phase SNP selection procedure used in this pipeline is necessary because when applied to call SNPs in the first phase, VarScan returns a reference value when a position fails any of the filters, and hence not enough information is present to call a state. During the second phase, all relevant positions are re-evaluated to correct those which have erroneously been identified as reference values instead of missing values (N). The determined nucleotide states at all of the listed positions were used to build a SNP matrix. The SNP matrix entries were concatenated per isolate to obtain a multiple FASTA file for phylogenetic analysis.

#### Adapted CFSAN SNP Pipeline-based subtyping workflow

The adapted CFSAN-based subtyping workflow included the same data analysis steps and parameters as the CFSAN-based workflow except that the minimal alternative allele frequency threshold applied during the first phase of the SNP selection procedure was changed from 100% to 90%, and the minimal alternative allele frequency threshold applied during the second step of the SNP filtering procedure was changed from 50% to 90%.

#### Reference genomes

The SNP-based subtyping procedures were carried out using two different closed reference genomes, namely *S*. Typhimurium LT2, which is most referred to in literature, and *S*. Typhimurium SL1344, which is described as reference genome for more virulent strains like *S*. Typhimurium ST313 [58,59]. Both reference genomes belong to ST-19, while the isolates from the tested dataset belong to ST-19 and ST-34. ST-19 and ST-34 make both part of the eBurstGroup 1, thus differing from each other only by an allele call on a single locus [60].

#### Phylogenetic trees

The phylogenies were inferred based on the obtained multiple FASTA files with MEGA7 [61]. Model selection for maximum-likelihood phylogeny was performed separately for each pipeline, and the optimal model was selected based on the Akaike information criterion (AIC) and the Bayesian information criterion (BIC, [62]). The maximum-likelihood phylogenetic trees were constructed with MEGA7-cc, using GTR model with uniform evolution rate (which performed optimally for each dataset based on AIC and BIC), with 100 bootstrap replicates and including all missing data sites (N).

#### SNP distance matrices

For CSI-, CFSAN- and adapted CFSAN-based workflows, the SNP distance matrices were provided as pipeline output. For the PHEnix- and adapted PHEnix-based workflows, the matrices were created using a custom script. Thereby, the SNP matrix positions were compared for each isolate pair, counting the number of equal recordings and ignoring positions with masked values (N). When tested on the multiple fasta files produced by the CSI-, CFSAN- and adapted CFSAN-based workflows, the script returned the same results as in the matrices provided in the pipeline output, thereby ruling out that the differences observed between the workflows were due to differences in distance calculation.

#### Down-sampling

To generate the down-sampled dataset, reads were mapped on the LT2 reference genome using BWA 0.7.12 *mem*, and the average coverage was calculated for each sample using the *multi-sample BAM QC* function of Qualimap 2.2.1. Based on the obtained coverage values, a fraction of original reads was randomly selected for each isolate to obtain a depth of 30X (S1 File). The resulting data was analysed with Qualimap to confirm that the down-sampling procedure did not disturb the original distribution of the reads across the reference genome.

#### Discriminatory power and epidemiologic concordance

To evaluate the workflows, we determined the epidemiologic concordance and the discriminatory power according to the same strategy as described by Gaia et al. [63]. The values were calculated using the Ridom EpiCompare 1.0 tool (http://www3.ridom.de/epicompare/). Epidemiologic concordance, defined as the fraction of pairs of epidemiologically related isolates that were assigned to the same type [22], was calculated as the Wallace’s coefficient with the only subtyping category being the outbreak isolates [64]. The index of discriminatory power was calculated as Simpson’s index of diversity [65,66]. For calculation of the discriminatory power index, the isolates were assigned to different subtypes as soon as they differed from each other by at least one SNP position included in the final SNP matrix. This approach, also applied by [22], allows to calculate the highest possible resolution that can be obtained with a particular workflow, without being bound by the on-going discussion about the genetic distance that is typically observed between isolates of the same subtypes.

## Results

### Comparison of the three SNP-based WGS typing pipelines using the original (high-coverage) dataset

Initially, the workflows were applied on the original dataset consisting of NGS data with an average coverage of 175X and with the LT2 reference genome. As this coverage is much higher than one would expect in a routine situation, this setting represents the ideal baseline situation.

#### Epidemiologic concordance and phylogenetic tree inference

**Fig 2:**
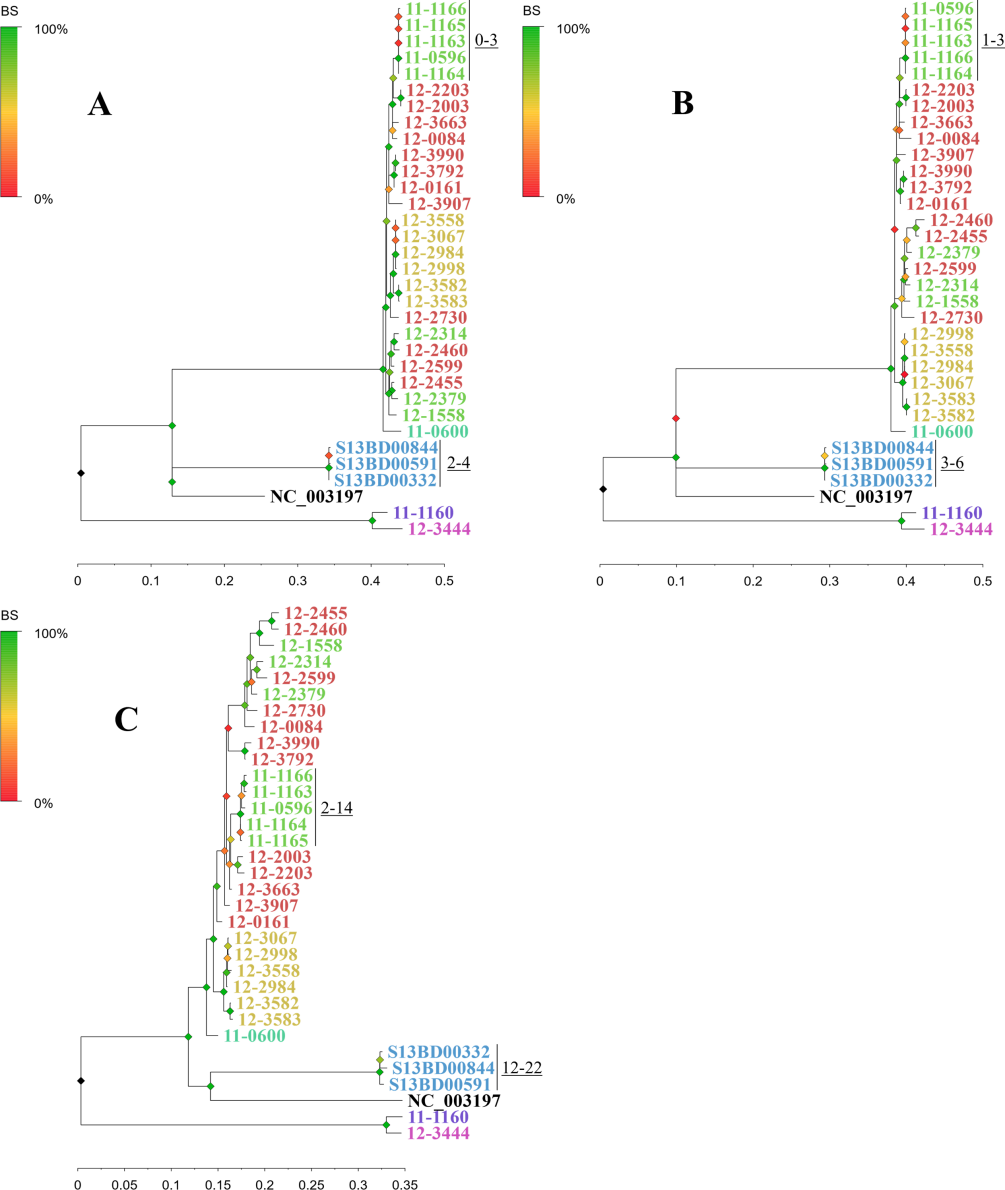
Phylogenetic tree generated with the tested SNP-based subtyping workflows. The workflows were run using the original dataset and LT2 as a reference genome. (A) CSI-based workflow, (B) PHEnix-based workflow, (C) CFSAN-based workflow. Isolates are coloured according to the MLVA-profile. The minimal and maximal SNP distances observed between the five outbreak isolates and the three isolates obtained from the same patient are indicated near the clusters. The two stably recurring groups of isolates mentioned in the text consist of (1) 12–3582, 12–3583, 12–2998, 12–2984, 12–3558, 12–3067 (yellow) and (2) 12–2314, 12–2460, 12–2599, 12–2455, 12–2379, 12–1558 (green and red). The trees are drawn to scale, with branch lengths measured in the number of substitutions per site. The scale axis is provided below each tree. BS: bootstrap values.

The consistency of the constructed phylogenetic trees was evaluated based on the correct co-clustering of the outbreak isolates, expressed as the epidemiologic concordance. Despite the different implementation specificities, the CSI-, PHEnix- and CFSAN-based workflows were all able to correctly categorise the outbreak isolates using the high coverage dataset, giving an epidemiologic concordance of 100% (Fig 2A–2C, Table 2). Also the isolates obtained from the same patient were as expected co-clustered by each of the three workflows. The phylogenetic trees showed considerable similarities, all retrieving besides the outbreak cluster two additional stable isolate clusters (Fig 2, Table 2). Small differences between the methods were observed in the exact positions of the isolates within the clusters, the relative positions of the clusters in the inferred trees, and the positions of some *S*. Typhimurium isolates with the 15-3-1 MOL-PCR profile.

**Table 2:** Performance metrics of the tested SNP-based subtyping workflows.

	CSI-based workflow		PHEnix-based workflow		CFSAN-based workflow	
* *	OD	30X	OD	30X	OD	30X
*Epidemiologic concordance*	100%	100%	100%	100%	100%	100%
*SNP matrix size*	999	537	1649	1056	2008	1732
*Number of subtypes*	28	23	29	28	32	32
*DP*	0.992	0.972	0.994	0.990	1.00	1.00
*Confidence interval of DP*	0.982–1.00	0.947–0.996	0.985–1.00	0.976–1.00	1.00–1.00	1.00–1.00
*SNPs outbreak isolates*	0–3	0–2	1–3	1–3	2–14	7–17
*SNPs isolates from one patient*	2–4	0	3–6	1–4	12–22	16–23

Performance metrics of the workflows were assessed using original dataset (OD) and dataset down-sampled to a 30X coverage (30X), with LT2 as a reference genome. DP: discriminative power. SNPs outbreak isolates: minimal and maximal number of SNPs observed between the outbreak isolates. SNPs isolates from one patient: minimal and maximal number of SNPs observed between isolates obtained from the same patient.

In contrast to the CSI-, PHEnix- and CFSAN-based workflows, in the phylogeny generated with the adapted PHEnix-based workflow, one of the outbreak isolates, 11–0596, did not make part of the outbreak cluster (S4 Fig), giving an epidemiologic concordance of 60% only. Also the placement of non-outbreak isolates was different compared to the CSI-, PHEnix- and CFSAN-based workflows. For instance, while the isolates 12–3990 and 12–2203 were closely clustered with respectively isolates 12–3792 and 12–2003 in the trees produced by the CSI-, PHEnix- and CFSAN-based workflows, in the tree created with the adapted PHEnix-based workflow, the two isolates were placed near the root of the tree together with 11–0596. Remarkably, these isolates that were placed together by the adapted PHEnix-based workflow i.e. 12–3990, 12–2203 and 11–0596 were the ones that showed an uneven coverage as elaborated in the quality control section of the Materials and Methods.

#### Discriminatory power and SNP distances between isolates

**Fig 3:**
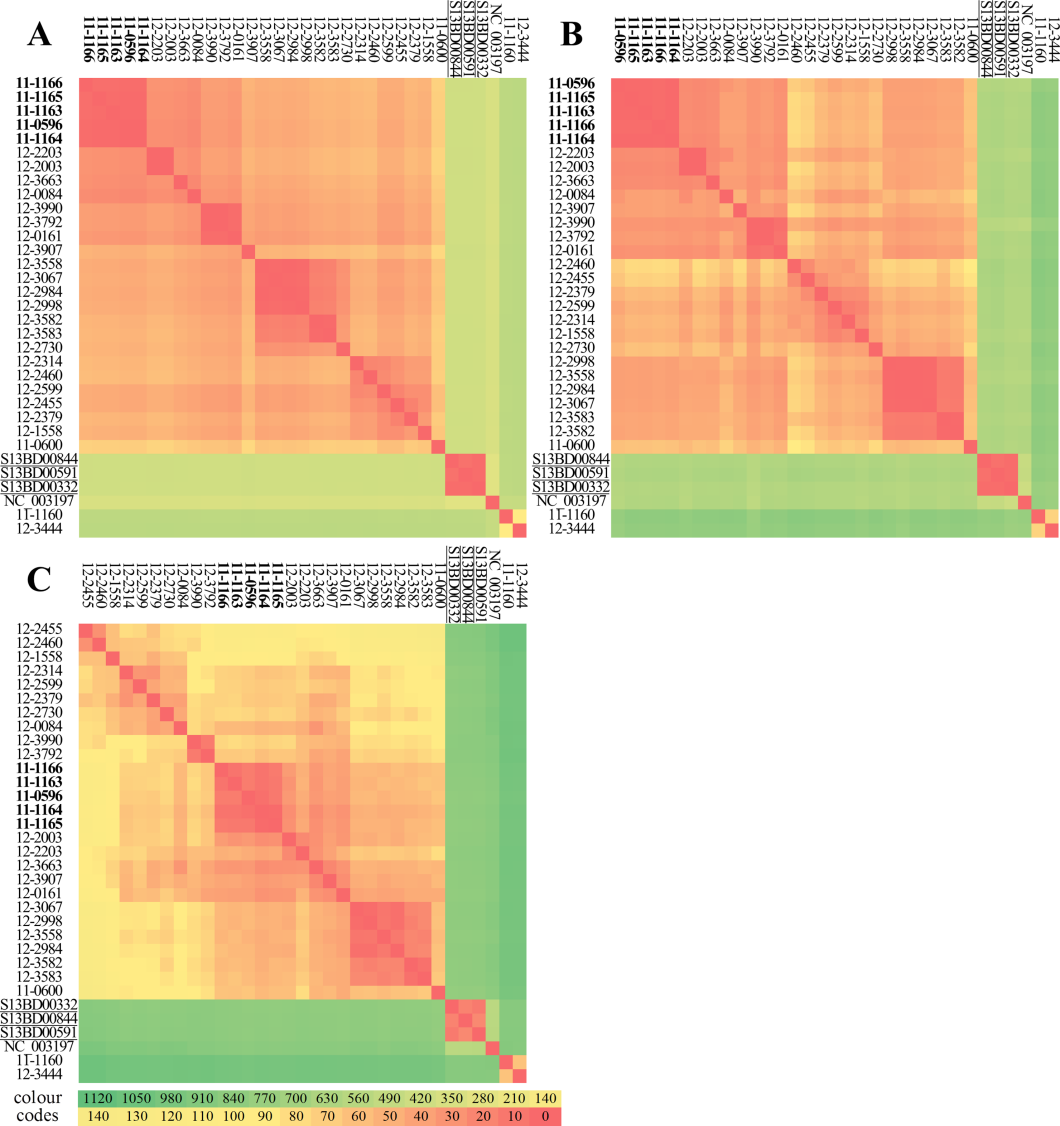
SNP distance matrices generated with the tested SNP-based subtyping workflows. The workflows were run using the original dataset and LT2 as a reference genome. (A) CSI Phylogeny-based workflow, (B) PHEnix-based workflow, (C) CFSAN-based workflow. Colours indicate pairwise SNP distances between isolates. Outbreak isolates are shown in bold and isolates obtained from the same patient are underlined.

The CFSAN-based workflow retained the highest number of polymorphic sites, followed by the PHEnix- and the CSI-based workflows (Table 2, Fig 3). Concordantly, the CFSAN-based workflow was able to discriminate between all 32 analysed isolates, whereas for PHEnix- and the CSI-based workflows a slightly lower number of observed subtypes was distinguished, and therefore a lower discriminatory power was observed (Table 2). The decrease in discriminatory power was however relatively low, and the overlapping confidence intervals of the discriminative power indices indicated that inferred differences between the discriminative power coefficients were not statistically significant.

Examination of the number of SNP positions returned by each pipeline at the various data analysis stages showed that the main differences between the PHEnix- and the CFSAN-based workflows were due to both more stringent variant selection and SNP matrix construction steps of the PHEnix-based workflow (S3 Fig, S1 Table). Because the CSI pipeline does not provide the output of the intermediate data analysis steps, the stringency of the variant selection and SNP matrix construction steps could not be compared with those of the other pipelines.

The three tested workflows returned highly different SNP distances between outbreak isolates (0–3, 1–3 and 2–14 SNPs for the CSI-, PHE- and CFSAN-based workflows, respectively) and between the isolates obtained from the same patient (2–4, 3–6 and 12–22 SNPs for the CSI-, PHE- and CFSAN-based workflows respectively) (Table 2, Fig 3). Besides the outbreak isolates, the benchmark dataset showed to contain additional isolate pairs with mutual SNP distances laying in the same range as the intra-outbreak SNP distances (Fig 3 and S7 Fig). Most of such isolate pairs, including for instance the first (S13BD00332) and the second (S13BD00591) isolate obtained from the same patient, were retrieved by all three workflows (with 2, 3 and 12 SNPs for the CSI-, PHE- and CFSAN-based workflows, respectively). However, for some of such closely related isolate pairs the output of the workflows disagreed on the inferred genetic distance relative to the inter-outbreak isolates, for example for 12–0161 and 12–3792 (2, 8 and 40 SNPs for the CSI-, PHE- and CFSAN-based workflows, respectively), for 12–0161 and 12–3990 (2, 2 and 50 SNPs for the CSI-, PHE- and CFSAN-based workflows, respectively) and to a lesser extent for the third (S13BD00844) and the first (S13BD00332) isolates obtained from the same patient (2, 3 and 22 SNPs for the CSI-, PHE- and CFSAN-based workflows, respectively).

### Sensitivity of the WGS typing workflows to decreasing coverage

To test if the small differences observed between the pipelines using the high-coverage dataset would become more pronounced using data with a lower coverage, we have down-sampled the original dataset to obtain an average sequencing depth of 30X for each isolate and repeated the analysis.

The epidemiologic concordance of the phylogenies generated by all the three subtyping workflows still was 100% (S5 Fig, Table 2), with all outbreak isolates being correctly attributed to an outbreak-specific cluster. Also the stably recurring (non-outbreak) clusters observed with the original dataset were retrieved with the down-sampled dataset by all three workflows, with the only exception being the inclusion of one extra isolate (12–2730) in one of such clusters by the CFSAN-based workflow. For all three typing workflows, the trees generated with the down-sampled dataset diverged slightly from those generated with the original dataset, but the majority of the differences was situated at the branches with low bootstrap support.

Related to the discriminatory power, the workflows showed larger differences in their sensitivity towards the coverage changes. The CSI-based workflow showed the highest decrease in the number of discriminated subtypes (being reduced from 28 to 23 with decreasing coverage), followed by the PHEnix-based workflow which attributed the 32 isolates to 28 subtypes instead of 29, and the CFSAN-based workflow which was still able to discern between all 32 isolates (Table 2).

The CSI-based pipeline showed the strongest reduction of the SNP matrix size (Table 2). Examination of the SNP matrices revealed that this led to a near collapse of the otherwise distinct isolate groups, with the distances between some isolate pairs dropping from 10–12 SNP positions to 4–6 positions only (S8 Fig). Exchange of the SNP matrix construction rules between the different workflows and comparison of the sizes of the resulting SNP matrices (PHEnix+CSI and CFSAN+CSI in S1 Table) showed that the increased sensitivity towards the coverage of the CSI-based workflow resulted from both more stringent SNP selection and SNP matrix construction.

While as expected the inter-isolate distances generally decreased for the low-coverage dataset for all workflows, counterintuitively the SNP distances between closely related isolates appeared to increase with decreasing coverage for the CFSAN-based workflow (Table 2, S8 Fig). The changes were in some cases large as from 3 to 17 SNPs. Detailed examination of the positions responsible for the differences between the datasets revealed that the differences mostly arose firstly because at some positions, the 50% allele frequency threshold utilized during the second phase of variant selection was more often met for only one of the isolates with low-coverage data while being either met or not met for both isolates with the high-coverage dataset. Secondly, for a smaller fraction of the positions, isolate-specific SNPs were excluded from the SNP list with the high-coverage dataset because of having a sequencing error in one of the reads that mapped at that position, and thus not meeting the 100% allele frequency threshold of the first phase of variant selection.

Adapting the CFSAN-based workflow by changing the allele frequency thresholds to more optimal values alleviated the problem almost completely (S7 and S8 Figs). The adapted workflow showed a 100% epidemiologic concordance, and a discriminatory power close to that of the PHEnix-based pipeline with both the high and the low coverage datasets (S1 Table, S4, S5, S7 and S8 Figs). SNP distances between the outbreak isolates were 1–3 SNPs for both the high-coverage and the down-sampled datasets respectively, and the SNP distances between isolates from the same patient were 3–5 and 4–5 SNPs for the high-coverage and the down-sampled datasets, respectively.

### Estimation of the effect of the false positive SNPs on the calculation of discriminative power

In order to assess to what extent the calculation of the discriminative power could be biased by the appearance of false positive and/or false negative SNPs in the pipeline output, we have estimated the SNP distances that are reported for identical isolates based on simulated replicate datasets. Therefore, additional 30X datasets were created for five randomly chosen isolates (11–0596, 12–2203, 12–3582, 12–3792, S13BD00591) by repeating the down-sampling procedure. The initial 30X data of the 32 isolates was combined with the newly created data, and the analyses were re-run.

The CSI-and the PHEnix-based workflows detected a SNP distance of 0 SNPs for any of the five pairs of the simulated replicate isolates while the adapted CFSAN-based workflow detected a distance of 1 SNP for one pair of replicate isolates out of five (S9 Fig). The original CFSAN-based workflow in contrast reported relatively high distances between replicates, i.e. from 5 to 8 SNPs (S9 Fig). Taking into account this observation, for the CFSAN-based workflow the use of one SNP as threshold to discriminate between isolates thus likely resulted in a noticeable overestimation of its discriminatory power at 30X. Recalculating the discriminative power of the CFSAN-based workflow applied on the down-sampled dataset with 9 SNP (i.e. higher than the number of SNP different between replicates) positions as the minimal discrimination threshold would result in drop of the discriminatory power index to 0.996 (30 subtypes) which is indeed lower than what was observed at a discriminative threshold of 1.

### Sensitivity of the WGS typing workflows towards the choice of the reference genome

In order to compare the extent of the reference-specific effects between the tested subtyping workflows, the described analyses were repeated with a second reference genome, SL1344. The topologies of the phylogenetic trees differed considerably from those observed with the LT2 genome (S10–S12 Figs) for all of the tested workflows. While with the LT2 reference, the *S*. Typhimurium isolates with the 15-3-1 MOL-PCR profile were distributed throughout the tree, they were now attributed to one or two separate clusters. The recurring non-outbreak clusters discussed in the previous sections were stably retrieved by all pipelines and with both the original, and the down-sampled datasets. While the outbreak isolates were placed closely together in the trees generated with the CSI-, PHEnix-, CFSAN- and adapted CFSAN-based workflows, they were in some cases not co-clustered within the same branch but located hierarchically relative to each other close to the root of the tree. Because subtypes used for calculation of epidemiologic concordance were defined based on attribution of isolates to the same branch, such positioning of outbreak isolates would compromise the concordance values. However, the close relatedness of the outbreak isolates could still be clearly seen from the very short inter-isolate SNP distances produced by the three workflows (S13–S15 Figs). In the tree generated by the adapted PHEnix- based workflow, the genetic distances between the outbreak isolates, namely between the 11–0596 and the other four, were considerably higher. Despite the fact that all isolates showed a higher number of SNPs with SL1344 as a reference genome, the observations on the discriminatory power made for the different pipelines and the two analysed datasets were similar for both reference genomes (S2 Table).

## Discussion

Whole genome sequencing (WGS) is considered a very promising technology for subtyping of bacterial isolates, and because of the decreasing costs and improving technology, it is predicted to become an alternative to the subtyping methods currently applied in routine. Extraction of subtyping information from WGS data, however, is still challenging, and emerging workflows vary in their efficiency, discriminatory power, reproducibility, ease of interpretation and requirements for the raw read data. In this study, we have compared three different workflows for SNP-based subtyping of bacterial isolates using WGS data (original dataset and a down-sampled dataset with lower coverage) of 32 *S*. *enterica* isolates as a test case. The approaches were evaluated from the view point of a reference laboratory, and additional tests were carried out to identify the aspects of the data analysis process that were responsible for the observed inconsistencies between workflows.

From a technical point of view, all three tested workflows showed a 100% epidemiologic concordance. However, their discriminative power and the SNP distances between isolates appeared to be influenced differently by the sequencing coverage. These differences were associated with the stringency of the data analysis steps used by the subtyping schemes, and the resulting SNP matrix sizes. Indeed, the high coverage sensitivity of the CSI-based workflow appeared to be caused by the used SNP matrix construction strategy which excludes all positions for which no data was available for at least one of the isolates in combination with relatively stringent SNP selection parameters. While in theory the CSI Phylogeny pipeline allows to set more relaxed SNP selection parameters than the ones applied in this study, the SNP matrix construction rules cannot be changed. Also in several commercially available packages, the same SNP matrix construction rule is fixed to the most conservative choice (CLC Genomics Workbench, https://www.qiagenbioinformatics.com/), or suggested as a default (BioNumerics, http://www.applied-maths.com). However, both our study and Pettengill et al. [30] demonstrate that SNP matrices that include a certain low percentage of missing values perform better for phylogenetic inference, and might therefore be more preferred for routine subtyping workflows. To avoid confusion, it should be noted that in the study performed by Pettengill et al. [30], the term SNP matrix construction is used differently than in the current study. In the study of Pettengill et al. [30] it defines only the final step of the procedure, in which the SNPs that have already undergone the population-wide SNP filtering are combined into a fasta matrix. The original CFSAN-based workflow showed high epidemiologic concordance values and a good discriminative power. However, because of the specificities of the SNP identification procedure a number of false positive and/or false negative SNPs were present in the output, resulting in an increase of the inter-isolate distances with a lowering coverage and a likely overestimation of the true discriminative power. This issue could be solved by adapting the alternative allele frequency threshold used during the two SNP selection phases. It should be remarked that new versions of the CFSAN SNP pipeline (starting form 0.7.0), which became available after completing the analysis, include an additional variant filtering option allowing to remove SNPs located too close to other SNPs or to contig edges. Tests performed with the latest version of the pipeline (1.0.1, data now shown) illustrated that such filters allow to remove a large fraction of positions, but not all, that arise due to recombination and read mapping artefacts at imperfect repeat regions, having a positive effect on the number of incorrect SNPs in the output of the pipeline. The adapted CFSAN workflow showed the smallest sensitivity to the coverage changes, while still providing the correct output, which was due to the least stringent parameters utilized during both the variant selection and the SNP matrix construction steps that were applied therein. Among others, the implemented two-step variant selection procedure used a minimal coverage threshold of only 2 reads for the second filtering step. We believe based on these results that the performance of more conventional pipelines that use a single-step SNP-selection procedure could also be improved by using a lower coverage threshold than the one usually applied, for instance 4–5 reads instead of 8–20 reads [27,33,37,38,67–69]. Also the original PHEnix-based workflow returned good results both with respect to epidemiologic concordance and the sensitivity to the changes of the sequencing coverage of the dataset. Besides the original PHEnix-based workflow, we have tested a custom modification of the workflow, the adapted PHEnix-based workflow, that utilized variation of a SNP matrix construction strategy according to which genomic positions that do not meet SNP filtering criteria, such as the minimal coverage, are considered as being equal to the reference values. This strategy has been applied in different studies including some that are relatively recent [53–56]. However, we illustrate that it can produce erroneous results for sequencing samples with slightly lower quality characteristics, and should therefore be avoided.

From the point of view of the potential end-users of the pipelines in public health institutes, i.e. taking into account the impact of the differences in parameter settings or analysis steps, the PHEnix-based and the adapted CFSAN-based workflows performed optimally for routine subtyping of *S*. Typhimurium and *S*. Typhimurium-like strains. They indeed returned the most reproducible and epidemiologically correct output at a high resolution for both high and low sequencing coverage datasets. The CSI-based workflow also performed well with respect to the clustering of the epidemiologically related isolates, and showed a good discriminative power at a higher sequencing depth, but the discriminative power of the workflow was strongly affected by decreasing coverage, as discussed above. The workflow would therefore pose more stringent requirements for the quality of the sequencing data, preventing the analysis of the samples with a lower quality or coverage that are occasionally generated in a routine situation. On itself, a slightly lower discriminative power is not an issue for the routine subtyping workflows, as long as the number of subtypes remains within a practical and useful range allowing to discriminate between the epidemiologically related and unrelated isolates which was the case for the CSI-based workflow. But a high variability of the SNP distances depending on the dataset, such as the ones observed for the CSI-based workflow (and also the counter-intuitive changes observed for the original CFSAN-based workflow), are in our regard very inconvenient, as they can disturb the correct interpretation of the results. In general, SNP distances between closely related isolates were highly different for the different subtyping schemes. For instance for the outbreak isolates, the SNP distances obtained from the CSI-based workflow were as small as 0–3 SNPs with the high-coverage dataset and 0–2 with the low-coverage dataset, while for the original CFSAN-based workflow, these values were as high as 2–14 and 7–17 SNPs, respectively. Similarly, for the isolates obtained from the same patient, the CSI-based workflow returned 2–4 SNPs with the high-coverage dataset and 0 SNPs with the low coverage dataset, while the CFSAN-based workflow returned 12–22 and 16–23 SNPs, respectively. These observations imply that it will not be possible to define a single cut-off value for delineation of an outbreak as it has been attempted in some studies [37,70]. For each workflow, the threshold will need to be estimated separately and the variability of the observed SNP distances depending on the analysis settings will need to be investigated, and should be taken into account in the decision-making process.

Finally, the tested workflows differ substantially in their implementation and the ease of use, which is also an important factor for the end-users. The CSI Phylogeny pipeline is an online tool with a graphical interface, which makes it very user-friendly. This is clearly an advantage for non-bioinformatics expert users. The PHEnix and the CFSAN SNP pipelines, by contrast, need to be installed locally in a Linux environment, and are command-line based. Therefore, they require a certain level of bioinformatics expertise to use. While local installation and computational resources that are needed to run the PHEnix and the CFSAN SNP pipelines might be regarded as a disadvantage, they also imply that the analysis will be carried out in a controlled environment and will not depend on the availability of an external service. Regarding these considerations, we feel that the CSI Phylogeny pipeline is suitable when SNP-based subtyping analyses have to be carried out occasionally, while the other two workflows could despite the high adoption threshold be considered as a more suitable option in case a more frequent use is envisaged.

Conclusively, our study allowed selecting the approaches that are most suitable for routine subtyping of *S*. Typhimurium and *S*. 1,4,[5],12:i:- in a public health institution. The obtained knowledge can be used for the optimisation of SNP-based subtyping workflows of other pathogenic species.

## Supporting information

S1 Fig Comparison of the sequencing samples based on the read mapping statistics. Read mapping statistics were obtained from Qualimap reports of the raw reads mapped on LT2 and SL1344 reference genomes, and re-plotted in R to improve visualization.
(TIFF)

S2 Fig Original genome coverage plots generated by Qualimap with LT2 and SL1344 reference genomes. (TIF)

S3 Fig Comparison of CFSAN and PHEnix variant selection procedures. (TIFF)

S4 Fig Phylogenetic trees generated with the tested SNP-based subtyping workflows using high-coverage dataset and LT2 as a reference genome. (A) CSI-based workflow, (B) PHEnix-based workflow, (C) adapted PHEnix-based workflow, (D) CFSAN-based workflow, (E) adapted CFSAN-based workflow. Isolates are coloured according to the MLVA-profile. The minimal and maximal SNP distances observed between the five outbreak isolates and the three isolates obtained from the same patient are indicated near the clusters. The trees are drawn to scale, with branch lengths measured in the number of substitutions per site. The scale axis is provided below each tree. BS: bootstrap values.
(TIF)

S5 Fig Phylogenetic trees generated with the successful SNP-based subtyping workflows using down-sampled dataset and LT2 as a reference genome. (A) CSI-based workflow, (B) PHEnix-based workflow, (C) CFSAN-based workflow, (D) adapted CFSAN-based workflow. Isolates are coloured according to the MLVA-profile. The minimal and maximal SNP distances observed between the five outbreak isolates and the three isolates obtained from the same patient are indicated near the clusters. The trees are drawn to scale, with branch lengths measured in the number of substitutions per site. The scale axis is provided below each tree. BS: bootstrap values.
(TIF)

S6 Fig Phylogenetic trees generated with the successful SNP-based subtyping workflows using down-sampled dataset supplemented with replicate data and LT2 as a reference genome. (A) CSI-based workflow, (B) PHEnix-based workflow, (C) CFSAN-based workflow, (D) adapted CFSAN-based workflow. The minimal and maximal SNP distances observed between the five outbreak isolates and the three isolates obtained from the same patient are indicated near the clusters. The trees are drawn to scale, with branch lengths measured in the number of substitutions per site. The scale axis is provided below each tree. BS: bootstrap values.
(TIF)

S7 Fig SNP distance matrices generated with the tested SNP-based subtyping workflows using high-coverage dataset and LT2 as a reference genome. (A) CSI-based workflow, (B) PHEnix-based workflow, (C) adapted PHEnix-based workflow, (D) CFSAN-based workflow, (E) adapted CFSAN-based workflow. Values and colour codes in the SNP distance matrices indicate pairwise SNP distances between isolates. Outbreak isolates are shown in bold and isolates obtained from the same patient are underlined.
(TIF)

S8 Fig SNP distance matrices generated with the successful SNP-based subtyping workflows using down-sampled dataset and LT2 as a reference genome. (A) CSI-based workflow, (B) PHEnix-based workflow, (C) CFSAN-based workflow, (D) adapted CFSAN-based workflow. Values and colour codes in the SNP distance matrices indicate pairwise SNP distances between isolates. Outbreak isolates are shown in bold and isolates obtained from the same patient are underlined. For the CSI-based workflow, the distances between isolates 12–3582 and 12–3583 versus isolates 12–2984, 12–2998, 12–3067 and 12–3558 dropped from 10–12 SNP positions observed with the normal (high-coverage) dataset to 4–6 positions with the down-sampled dataset. For the CFSAN-based workflow, the distances between isolates 12–2984, 12–2998, 12–3067 and 12–3558 increased strongly (as far as from 3 to 17 SNPs) with the down-sampled dataset compared to the original data.
(TIF)

S9 Fig SNP distance matrices generated with the successful SNP-based subtyping workflows using down-sampled dataset supplemented with replicate data and LT2 as a reference genome. (A) CSI-based workflow, (B) PHEnix-based workflow, (C) CFSAN-based workflow, (D) adapted CFSAN-based workflow. Values and colour codes in the SNP distance matrices indicate pairwise SNP distances between isolates. Outbreak isolates are shown in bold and isolates obtained from the same patient are underlined.
(TIF)

S10 Fig Phylogenetic trees generated with the tested SNP-based subtyping workflows using high-coverage dataset and Sl1344 as a reference genome. (A) CSI-based workflow, (B) PHEnix-based workflow, (C) adapted PHEnix-based workflow, (D) CFSAN-based workflow, (E) adapted CFSAN-based workflow. The minimal and maximal SNP distances observed between the five outbreak isolates and the three isolates obtained from the same patient are indicated near the clusters. The trees are drawn to scale, with branch lengths measured in the number of substitutions per site. The scale axis is provided below each tree. BS: bootstrap values.
(TIF)

S11 Fig Phylogenetic trees generated with the successful SNP-based subtyping workflows using down-sampled dataset and SL1344 as a reference genome. (A) CSI-based workflow, (B) PHEnix-based workflow, (C) CFSAN-based workflow, (D) adapted CFSAN-based workflow. The minimal and maximal SNP distances observed between the five outbreak isolates and the three isolates obtained from the same patient are indicated near the clusters. The trees are drawn to scale, with branch lengths measured in the number of substitutions per site. The scale axis is provided below each tree. BS: bootstrap values.
(TIF)

S12 Fig Phylogenetic trees generated with the successful SNP-based subtyping workflows using down-sampled dataset supplemented with replicate data and SL1344 as a reference genome. (A) CSI-based workflow, (B) PHEnix-based workflow, (C) CFSAN-based workflow, (D) adapted CFSAN-based workflow. The minimal and maximal SNP distances observed between the five outbreak isolates and the three isolates obtained from the same patient are indicated near the clusters. The trees are drawn to scale, with branch lengths measured in the number of substitutions per site. The scale axis is provided below each tree. BS: bootstrap values.
(TIF)

S13 Fig SNP distance matrices generated with the tested SNP-based subtyping workflows using high-coverage dataset and SL1344 as a reference genome. (A) CSI-based workflow, (B) PHEnix-based workflow, (C) adapted PHEnix-based workflow, (D) CFSAN-based workflow, (E) adapted CFSAN-based workflow. Values and colour codes in the SNP distance matrices indicate pairwise SNP distances between isolates. Outbreak isolates are shown in bold and isolates obtained from the same patient are underlined.
(TIF)

S14 Fig SNP distance matrices generated with the successful SNP-based subtyping workflows using down-sampled dataset and SL1344 as a reference genome. (A) CSI-based workflow, (B) PHEnix-based workflow, (C) CFSAN-based workflow, (D) adapted CFSAN-based workflow. Values and colour codes in the SNP distance matrices indicate pairwise SNP distances between isolates. Outbreak isolates are shown in bold and isolates obtained from the same patient are underlined.
(TIF)

S15 Fig SNP distance matrices generated with the successful SNP-based subtyping workflows using down-sampled dataset supplemented with replicate data and SL1344 as a reference genome. (A) CSI-based workflow, (B) PHEnix-based workflow, (C) CFSAN-based workflow, (D) adapted CFSAN-based workflow. Values and colour codes in the SNP distance matrices indicate pairwise SNP distances between isolates. Outbreak isolates are shown in bold and isolates obtained from the same patient are underlined.
(TIF)

S1 Table Performance metrics describing the output of tested SNP-based subtyping workflows and combinations thereof assessed using LT2 as a reference genome. Performance metrics of the workflows were measured using original dataset (OD) and dataset down-sampled to a 30X coverage (30X), with LT2 as a reference genome. ad. CFSAN-based workflow: adapted CFSAN-based workflow. PHEnix + CSI, PHEnix + CFSAN, etc.: refer to a combination of the variant calling rules from the first mentioned workflow with the SNP matrix construction rules of the second mentioned workflow. DP: discriminative power.
(DOCX)

S2 Table Performance metrics describing the output of tested SNP-based subtyping workflows and combinations thereof assessed using SL1344 as a reference genome. Performance metrics of the workflows were measured using original dataset (OD) and dataset down-sampled to a 30X coverage (30X), with SL1344 as a reference genome. ad. CFSAN-based workflow: adapted CFSAN-based workflow. PHEnix + CSI, PHEnix + CFSAN, etc.: refer to a combination of the variant calling rules from the first mentioned workflow with the SNP matrix construction rules of the second mentioned workflow. DP: discriminative power.
(DOCX)

S1 File Perl script used for down-sampling of the sequencing data. (DOCX)
