# Supplementary figures and images for: Comparison of SNP-based subtyping workflows for bacterial isolates using WGS data, applied to Salmonella enterica serotype Typhimurium and serotype 1,4,[5],12:i:-
Source: PLoS One. 2018 Feb 6;13(2):e0192504. doi: 10.1371/journal.pone.0192504 (PMC5800660; doi:10.1371/journal.pone.0192504)

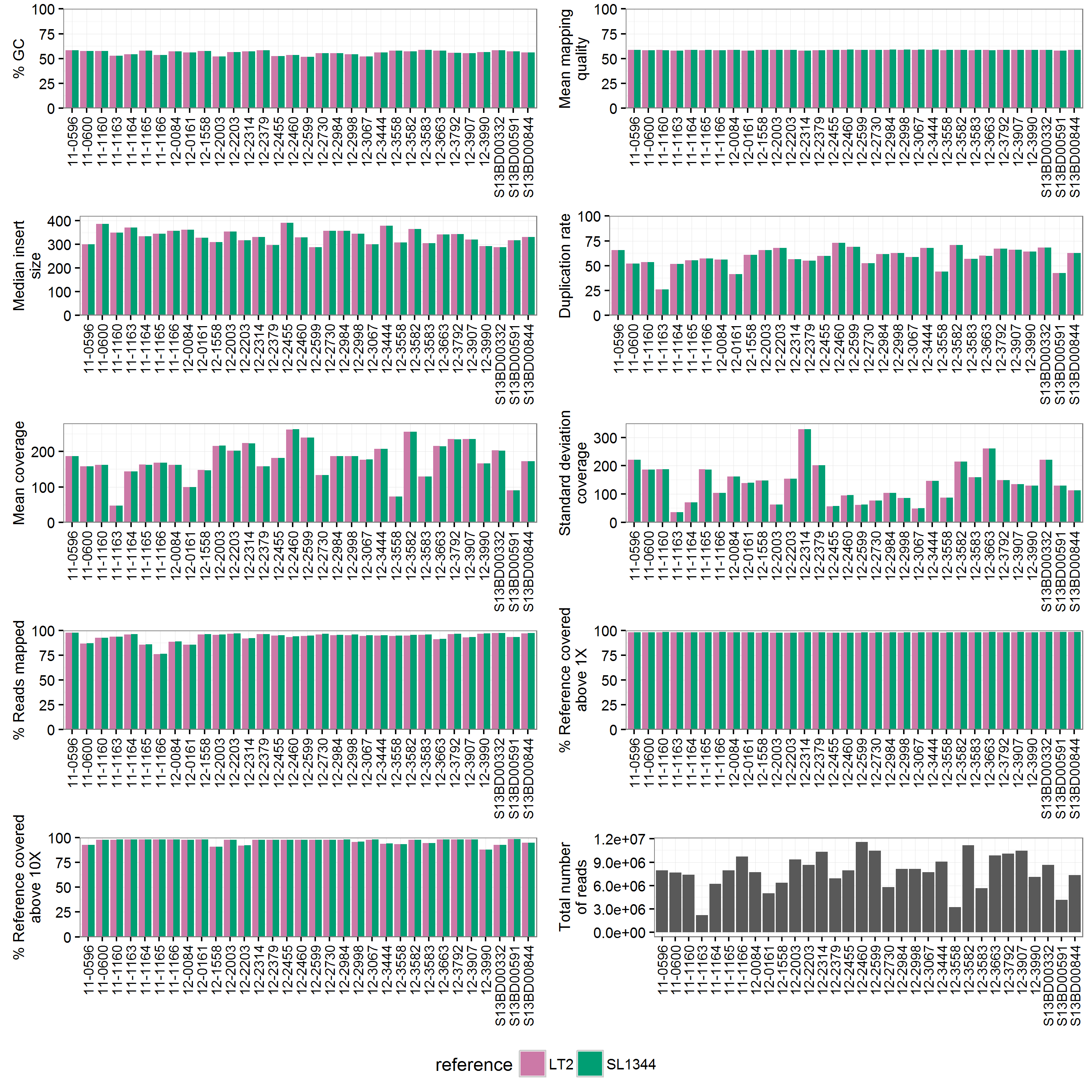

Supplement: S1 Fig — Read mapping statistics were obtained from Qualimap reports of the raw reads mapped on LT2 and SL1344 reference genomes, and re-plotted in R to improve visualization. (TIFF) [file pone.0192504.s001.tiff]

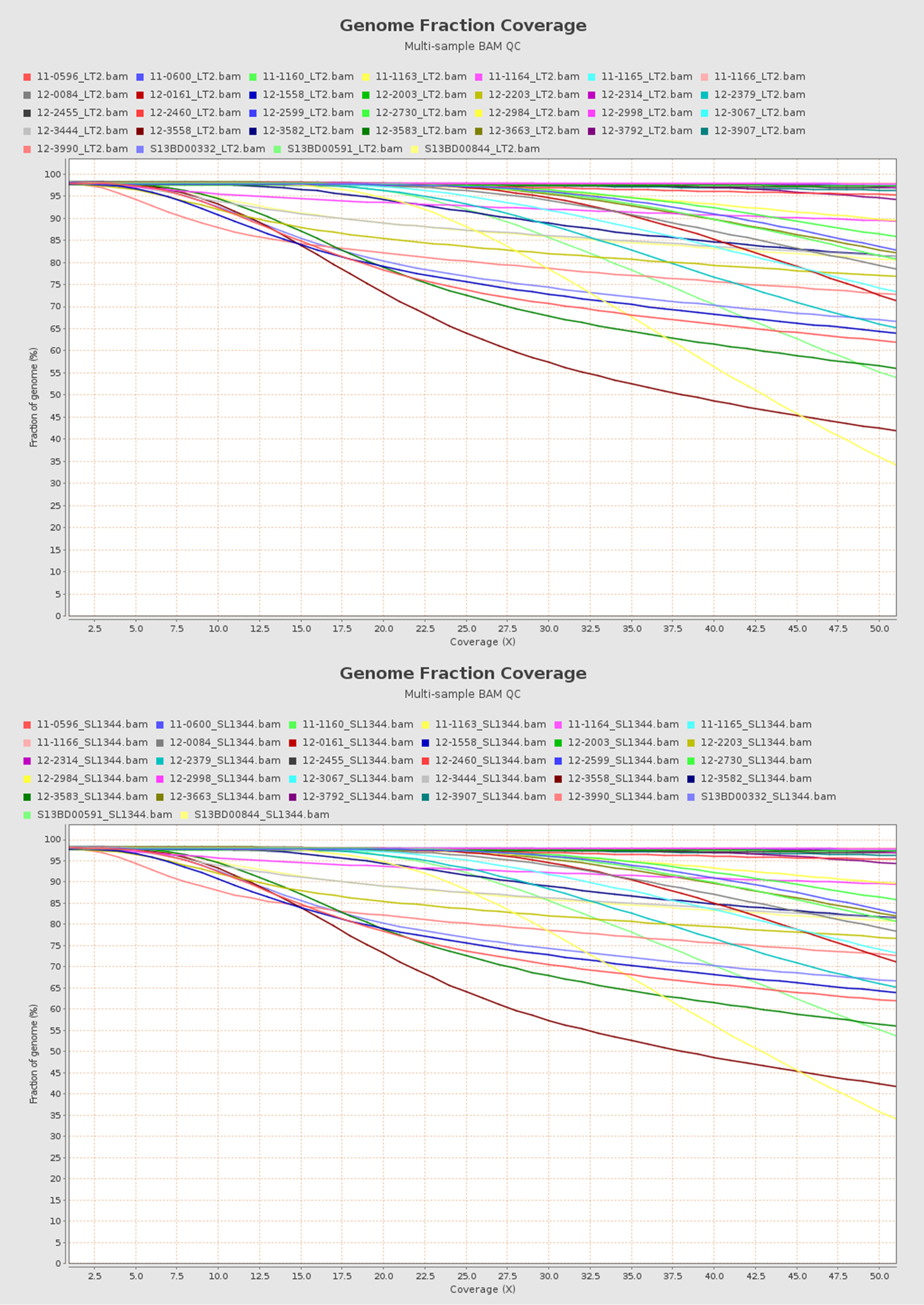

Supplement: S2 Fig — (TIF) [file pone.0192504.s002.tif]

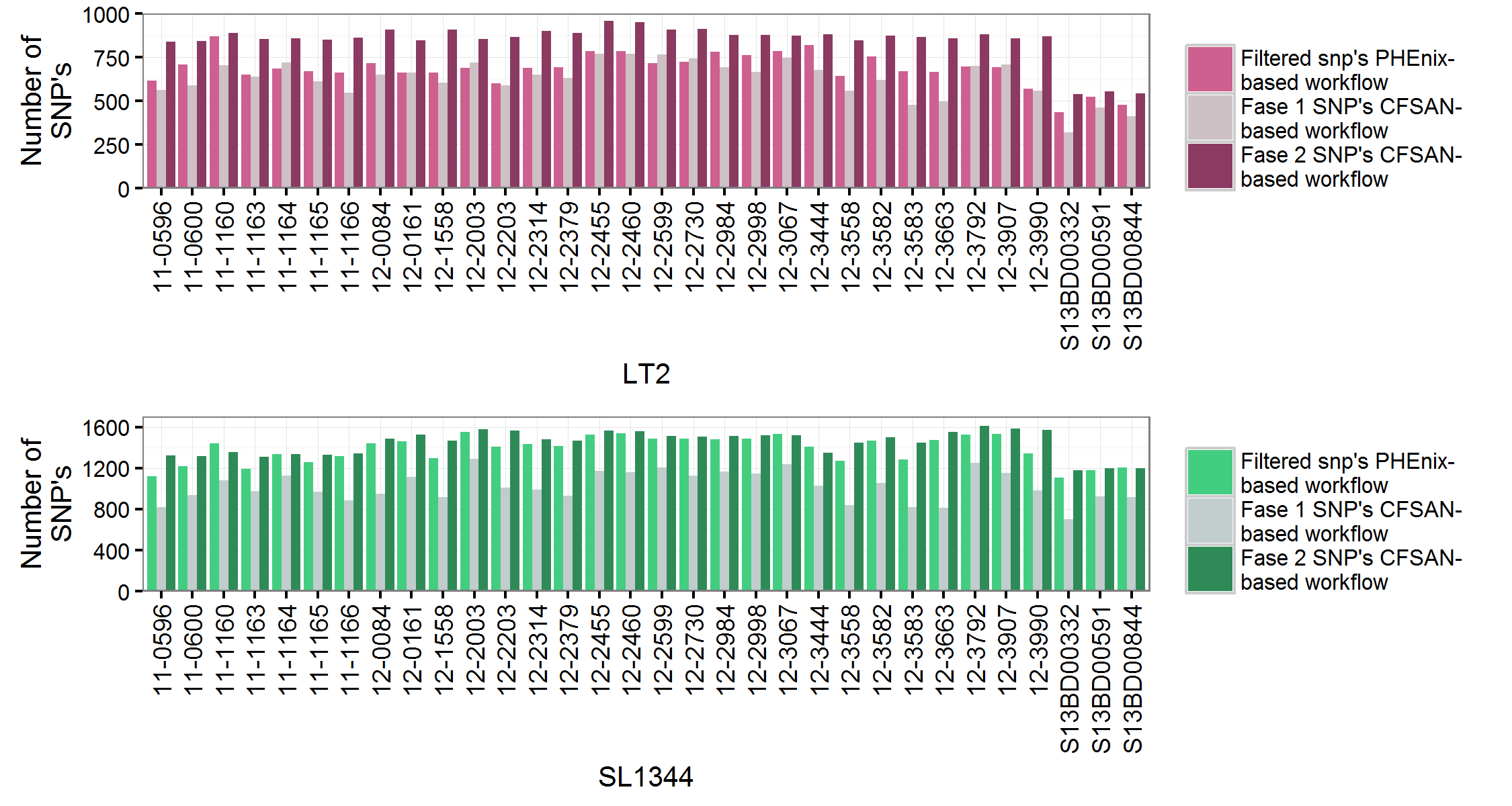

Supplement: S3 Fig — (TIFF) [file pone.0192504.s003.tiff]

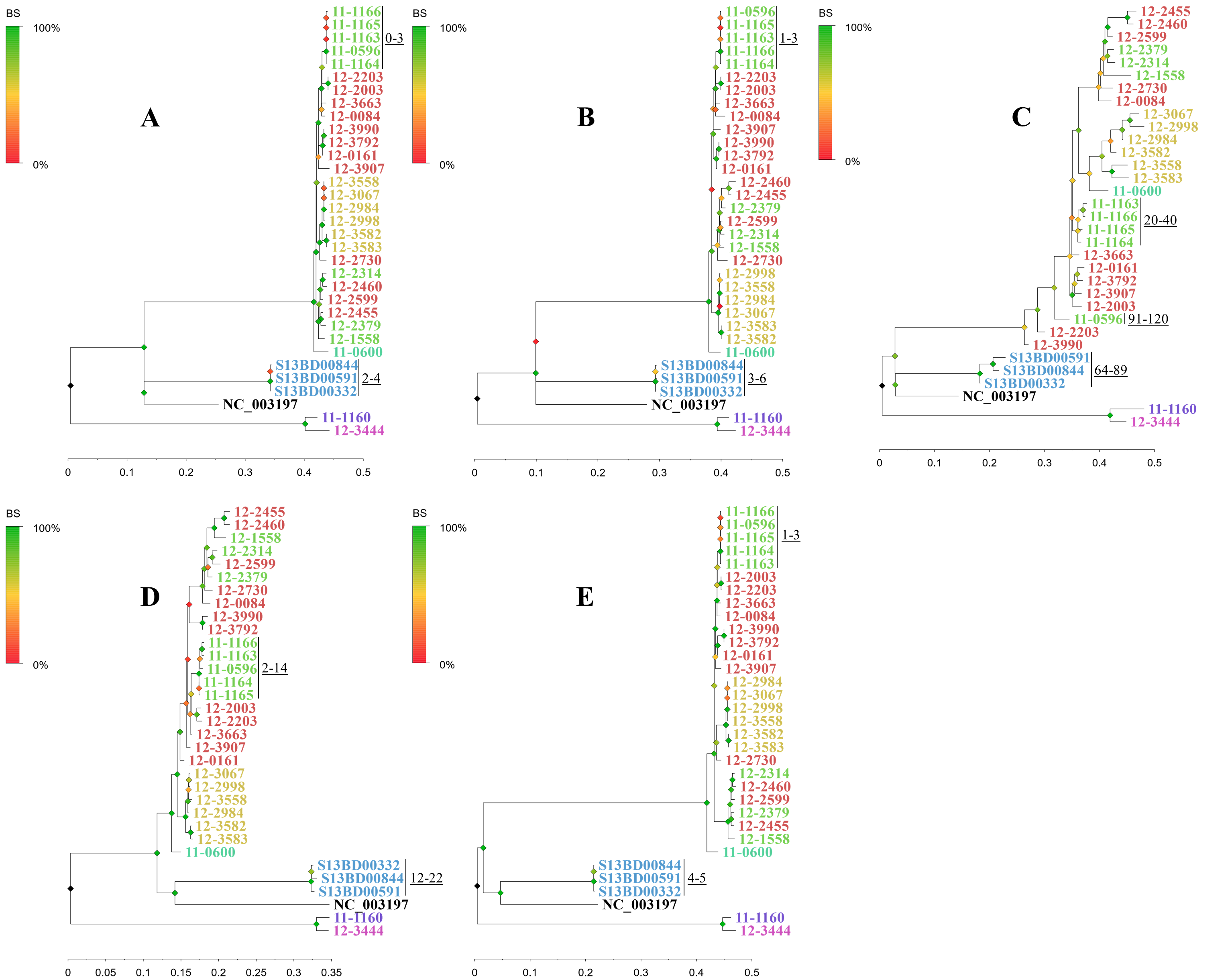

Supplement: S4 Fig — (A) CSI-based workflow, (B) PHEnix-based workflow, (C) adapted PHEnix-based workflow, (D) CFSAN-based workflow, (E) adapted CFSAN-based workflow. Isolates are coloured according to the MLVA-profile. The minimal and maximal SNP distances observed between the five outbreak isolates and the three isolates obtained from the same patient are indicated near the clusters. The trees are drawn to scale, with branch lengths measured in the number of substitutions per site. The scale axis is provided below each tree. BS: bootstrap values. (TIF) [file pone.0192504.s004.tif]

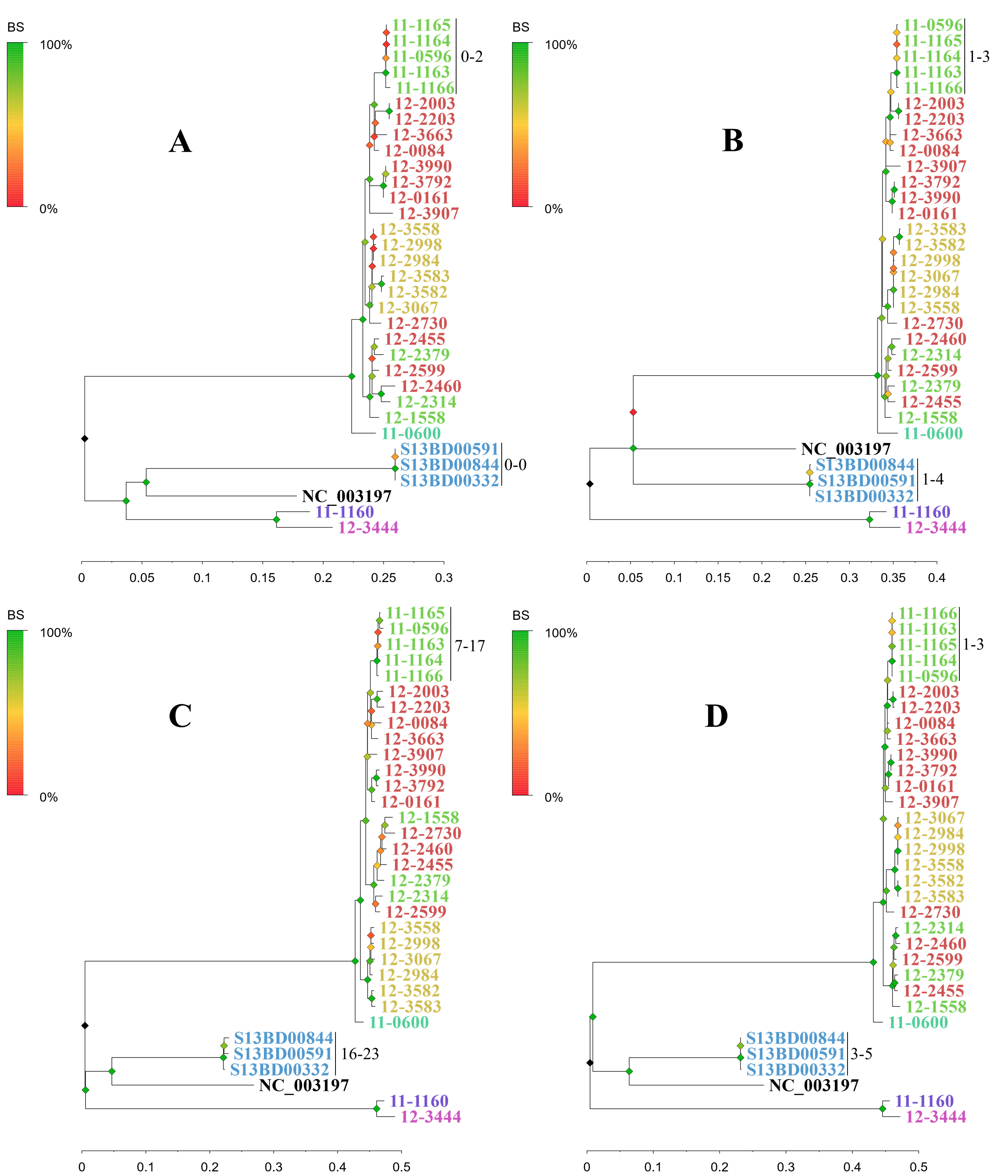

Supplement: S5 Fig — (A) CSI-based workflow, (B) PHEnix-based workflow, (C) CFSAN-based workflow, (D) adapted CFSAN-based workflow. Isolates are coloured according to the MLVA-profile. The minimal and maximal SNP distances observed between the five outbreak isolates and the three isolates obtained from the same patient are indicated near the clusters. The trees are drawn to scale, with branch lengths measured in the number of substitutions per site. The scale axis is provided below each tree. BS: bootstrap values. (TIF) [file pone.0192504.s005.tif]

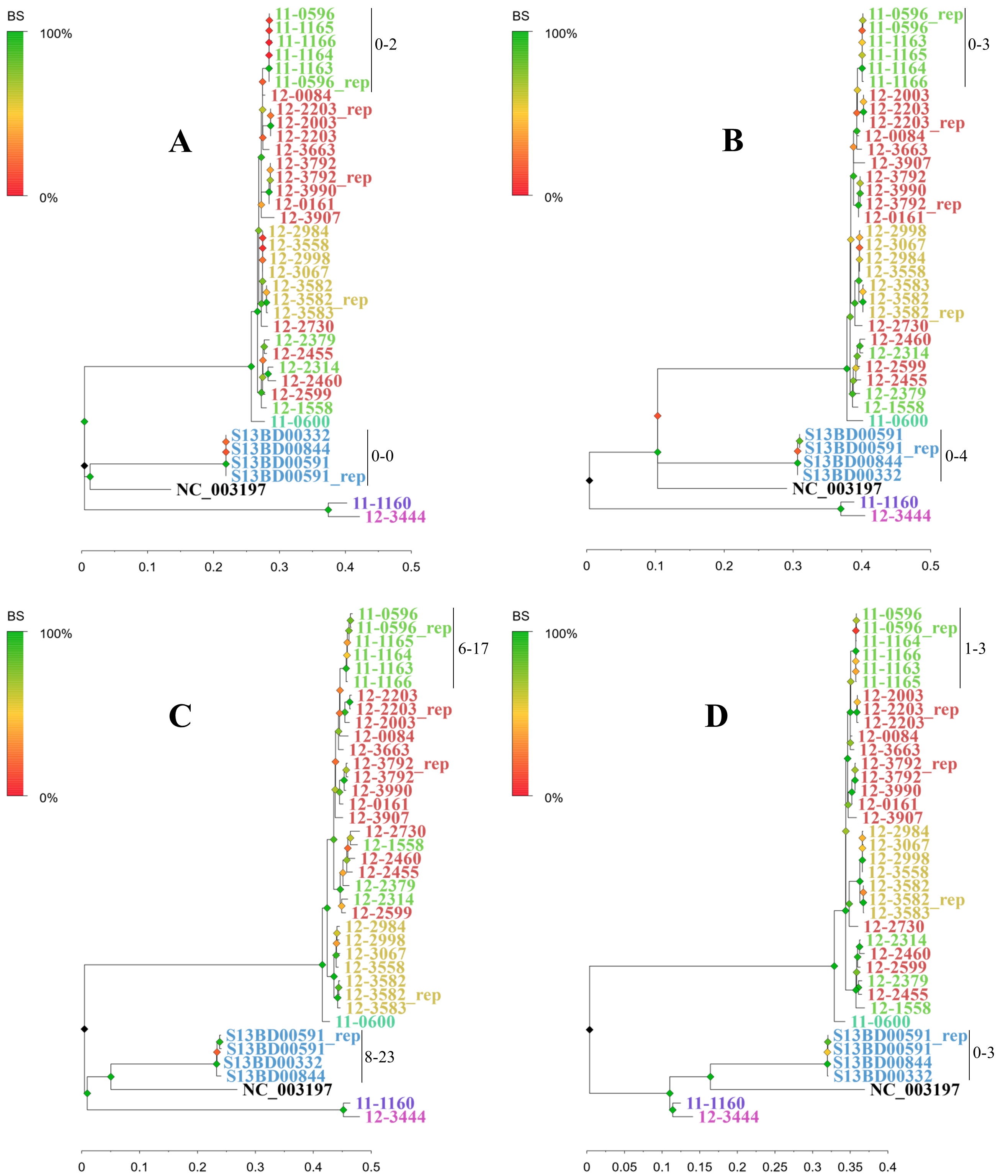

Supplement: S6 Fig — (A) CSI-based workflow, (B) PHEnix-based workflow, (C) CFSAN-based workflow, (D) adapted CFSAN-based workflow. The minimal and maximal SNP distances observed between the five outbreak isolates and the three isolates obtained from the same patient are indicated near the clusters. The trees are drawn to scale, with branch lengths measured in the number of substitutions per site. The scale axis is provided below each tree. BS: bootstrap values. (TIF) [file pone.0192504.s006.tif]

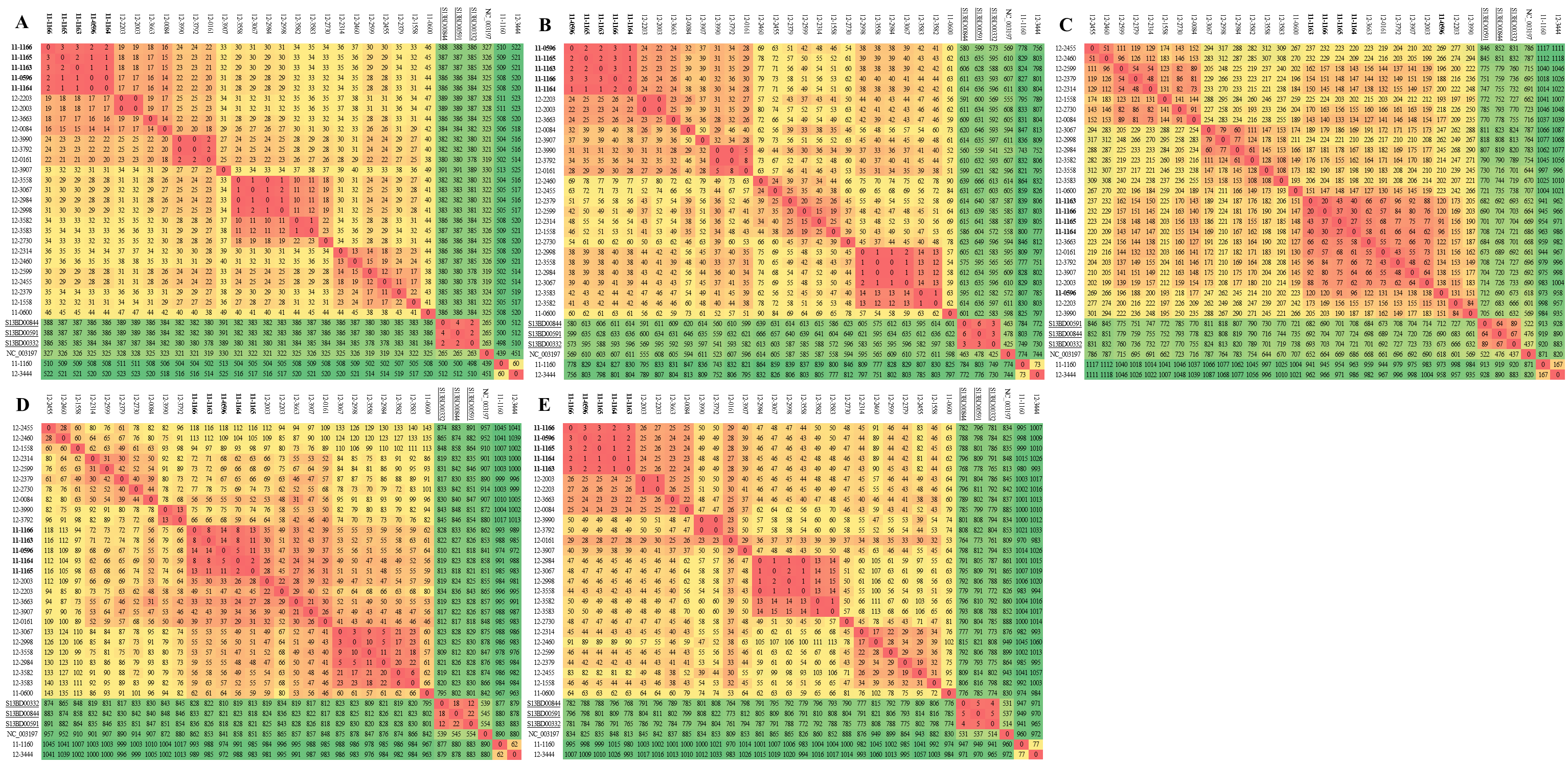

Supplement: S7 Fig — (A) CSI-based workflow, (B) PHEnix-based workflow, (C) adapted PHEnix-based workflow, (D) CFSAN-based workflow, (E) adapted CFSAN-based workflow. Values and colour codes in the SNP distance matrices indicate pairwise SNP distances between isolates. Outbreak isolates are shown in bold and isolates obtained from the same patient are underlined. (TIF) [file pone.0192504.s007.tif]

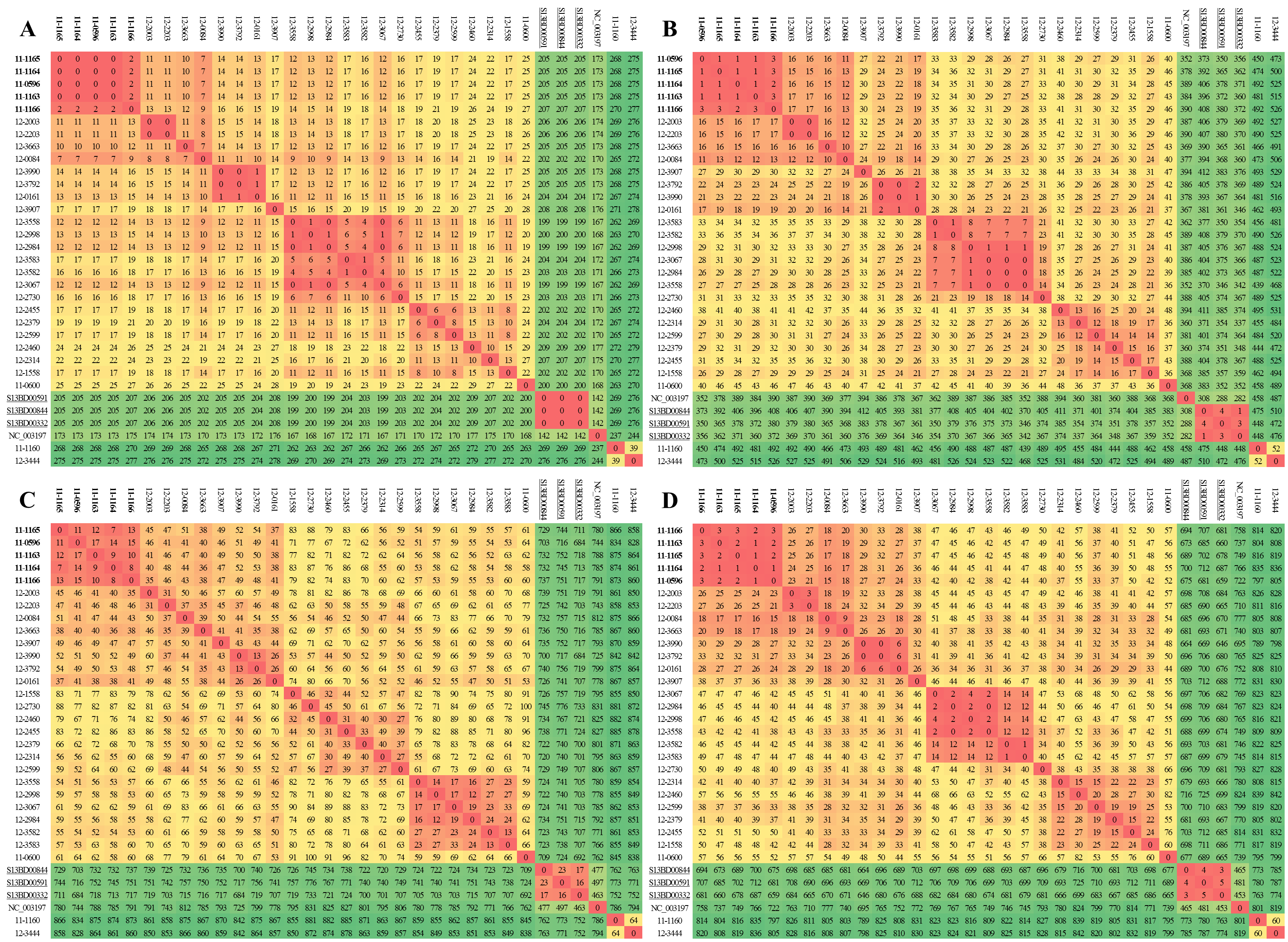

Supplement: S8 Fig — (A) CSI-based workflow, (B) PHEnix-based workflow, (C) CFSAN-based workflow, (D) adapted CFSAN-based workflow. Values and colour codes in the SNP distance matrices indicate pairwise SNP distances between isolates. Outbreak isolates are shown in bold and isolates obtained from the same patient are underlined. For the CSI-based workflow, the distances between isolates 12–3582 and 12–3583 versus isolates 12–2984, 12–2998, 12–3067 and 12–3558 dropped from 10–12 SNP positions observed with the normal (high-coverage) dataset to 4–6 positions with the down-sampled dataset. For the CFSAN-based workflow, the distances between isolates 12–2984, 12–2998, 12–3067 and 12–3558 increased strongly (as far as from 3 to 17 SNPs) with the down-sampled dataset compared to the original data. (TIF) [file pone.0192504.s008.tif]

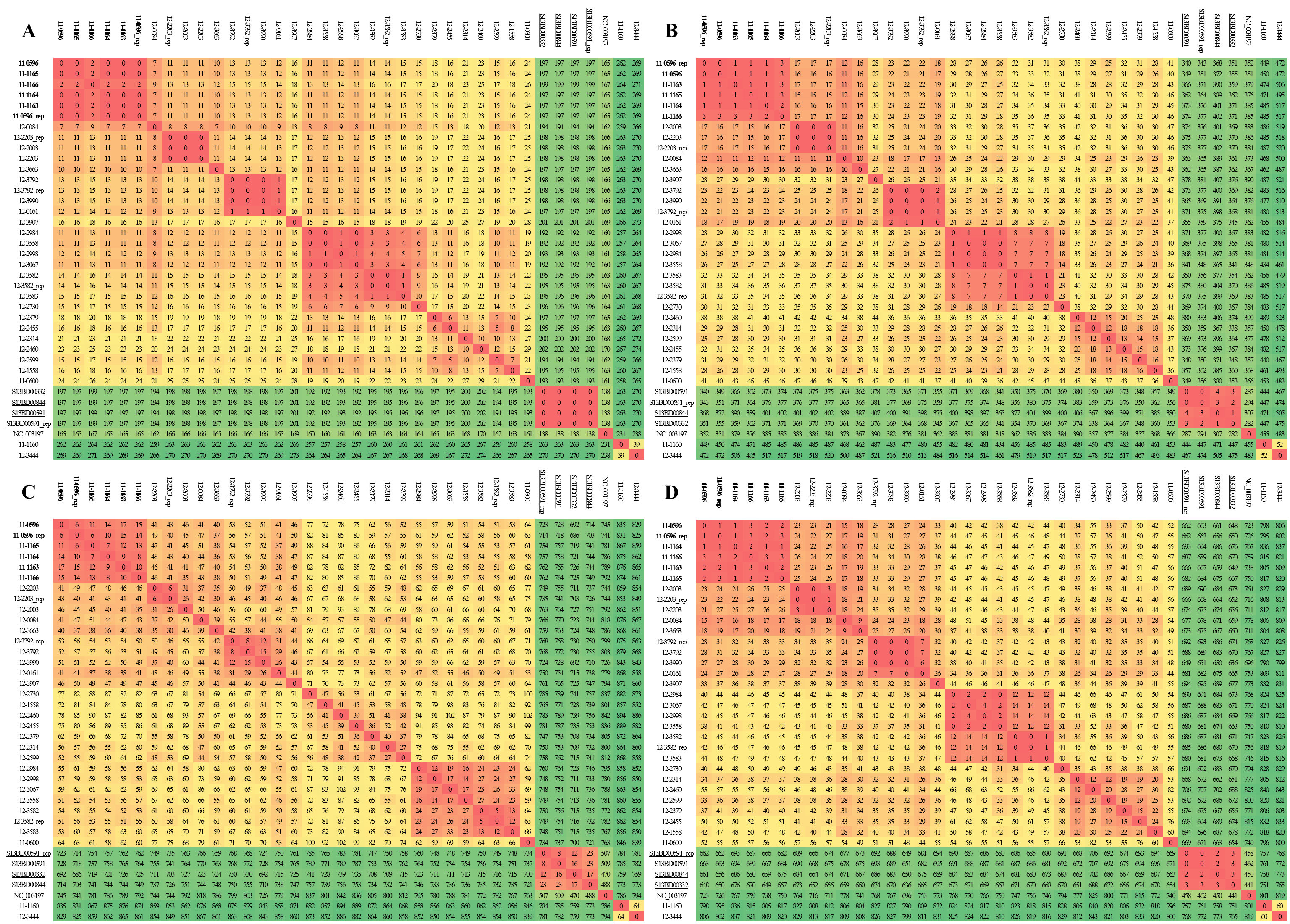

Supplement: S9 Fig — (A) CSI-based workflow, (B) PHEnix-based workflow, (C) CFSAN-based workflow, (D) adapted CFSAN-based workflow. Values and colour codes in the SNP distance matrices indicate pairwise SNP distances between isolates. Outbreak isolates are shown in bold and isolates obtained from the same patient are underlined. (TIF) [file pone.0192504.s009.tif]

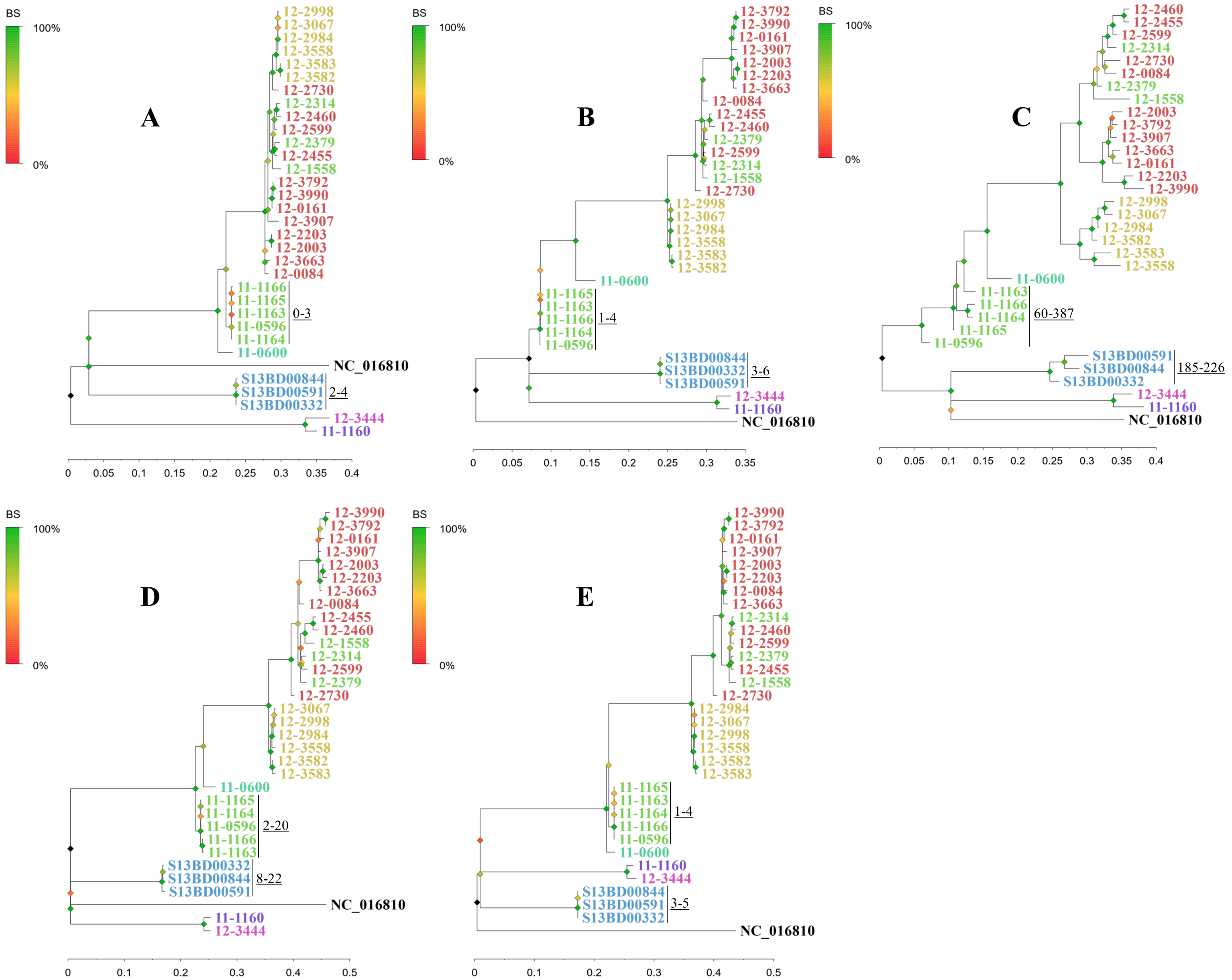

Supplement: S10 Fig — (A) CSI-based workflow, (B) PHEnix-based workflow, (C) adapted PHEnix-based workflow, (D) CFSAN-based workflow, (E) adapted CFSAN-based workflow. The minimal and maximal SNP distances observed between the five outbreak isolates and the three isolates obtained from the same patient are indicated near the clusters. The trees are drawn to scale, with branch lengths measured in the number of substitutions per site. The scale axis is provided below each tree. BS: bootstrap values. (TIF) [file pone.0192504.s010.tif]

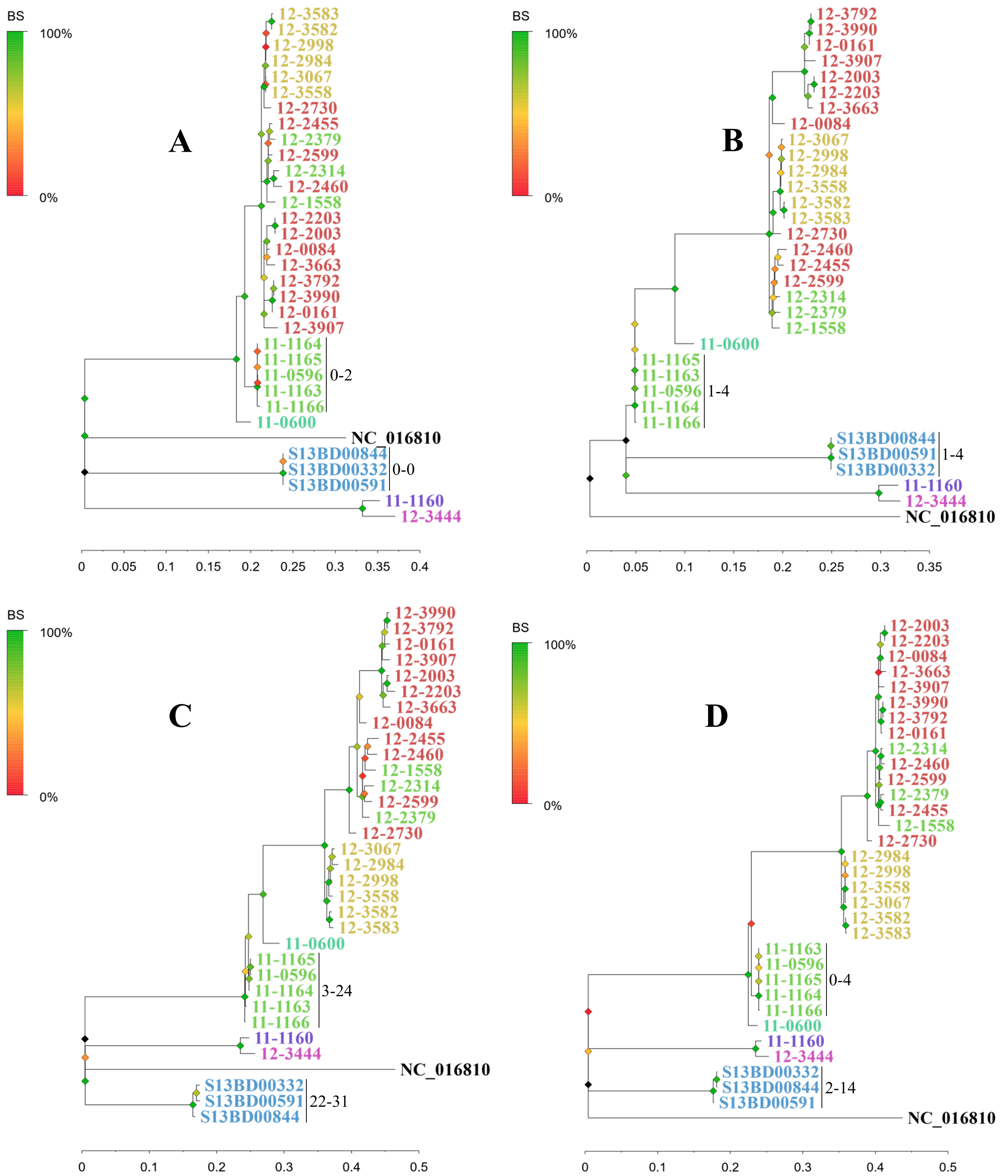

Supplement: S11 Fig — (A) CSI-based workflow, (B) PHEnix-based workflow, (C) CFSAN-based workflow, (D) adapted CFSAN-based workflow. The minimal and maximal SNP distances observed between the five outbreak isolates and the three isolates obtained from the same patient are indicated near the clusters. The trees are drawn to scale, with branch lengths measured in the number of substitutions per site. The scale axis is provided below each tree. BS: bootstrap values. (TIF) [file pone.0192504.s011.tif]

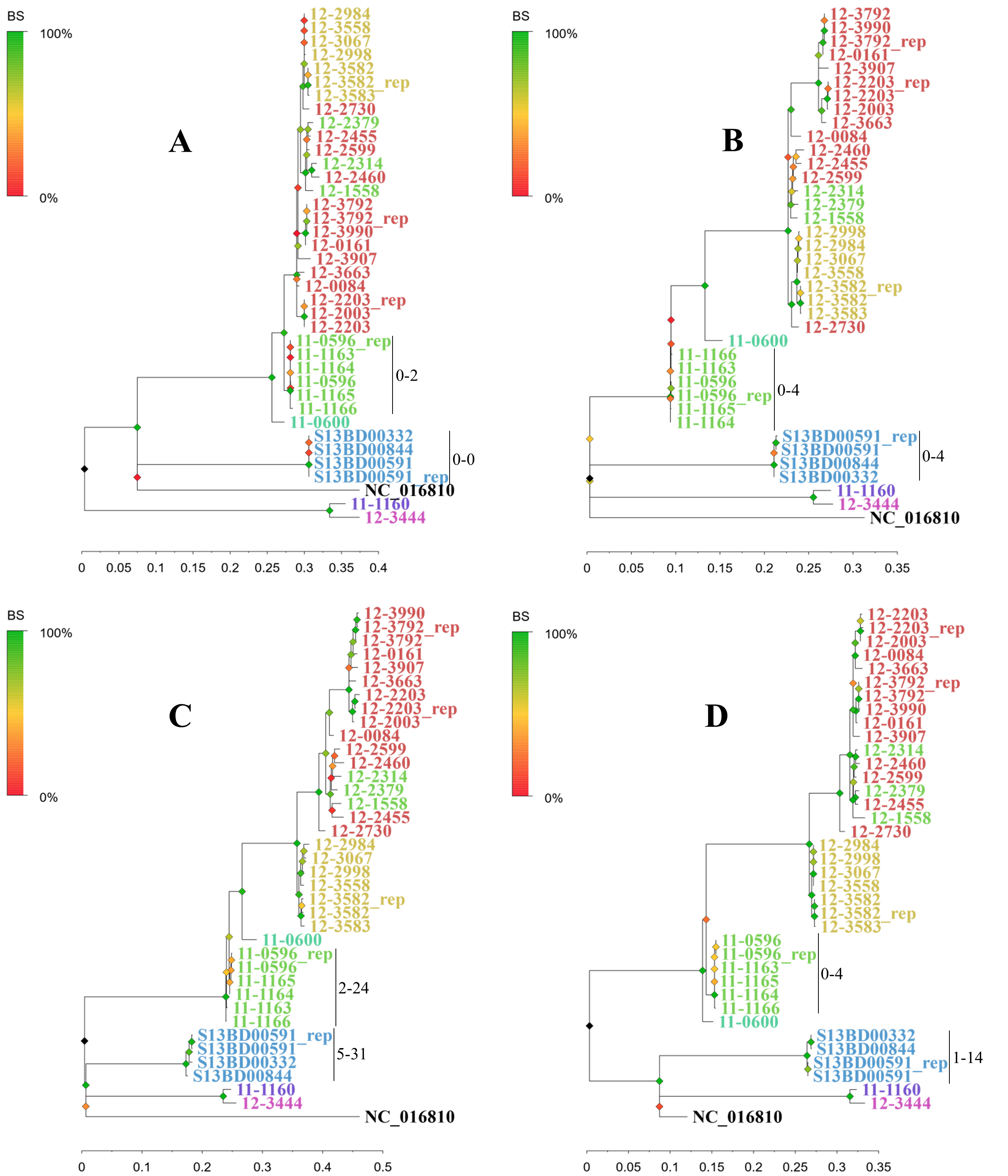

Supplement: S12 Fig — (A) CSI-based workflow, (B) PHEnix-based workflow, (C) CFSAN-based workflow, (D) adapted CFSAN-based workflow. The minimal and maximal SNP distances observed between the five outbreak isolates and the three isolates obtained from the same patient are indicated near the clusters. The trees are drawn to scale, with branch lengths measured in the number of substitutions per site. The scale axis is provided below each tree. BS: bootstrap values. (TIF) [file pone.0192504.s012.tif]

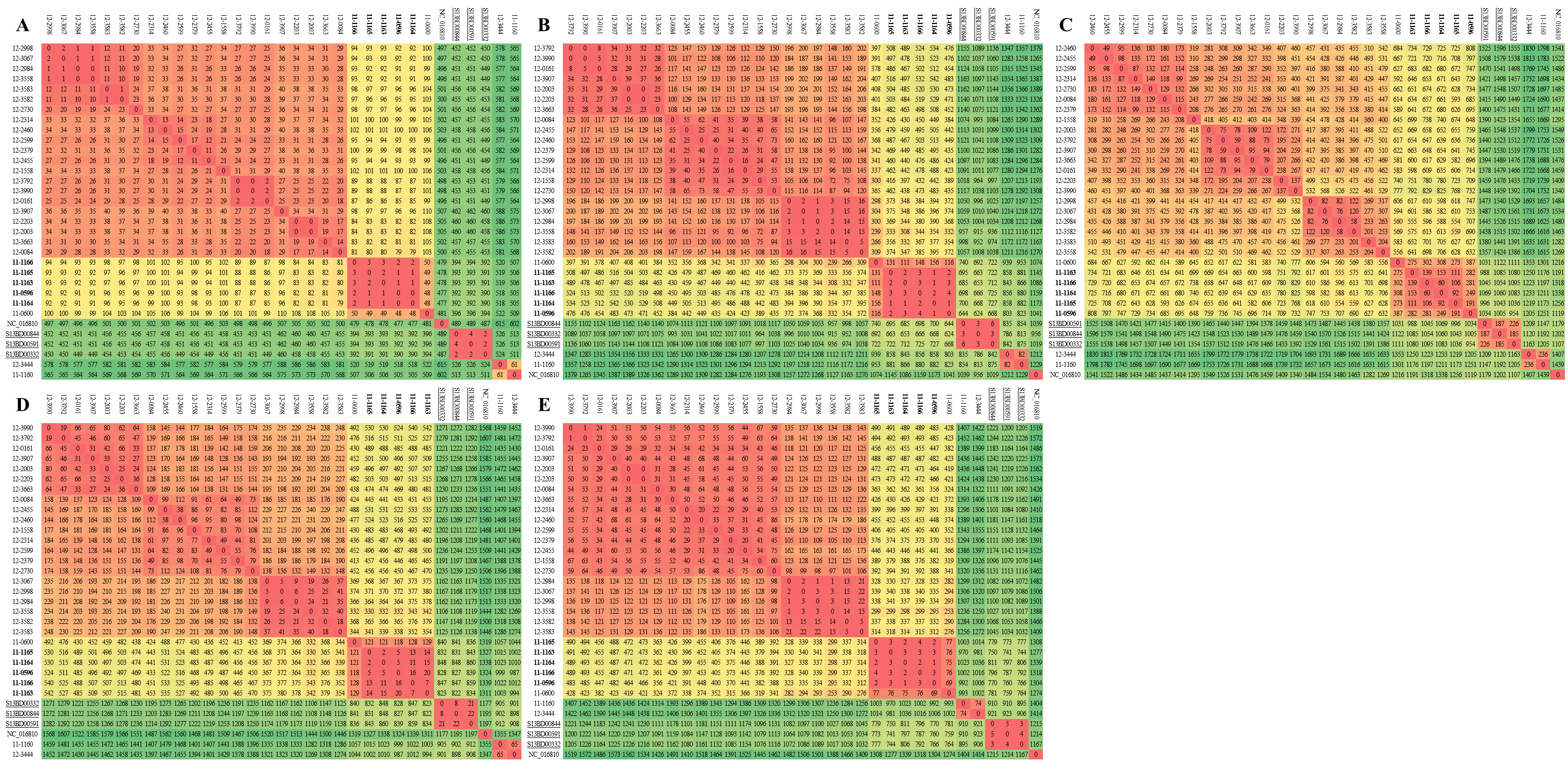

Supplement: S13 Fig — (A) CSI-based workflow, (B) PHEnix-based workflow, (C) adapted PHEnix-based workflow, (D) CFSAN-based workflow, (E) adapted CFSAN-based workflow. Values and colour codes in the SNP distance matrices indicate pairwise SNP distances between isolates. Outbreak isolates are shown in bold and isolates obtained from the same patient are underlined. (TIF) [file pone.0192504.s013.tif]

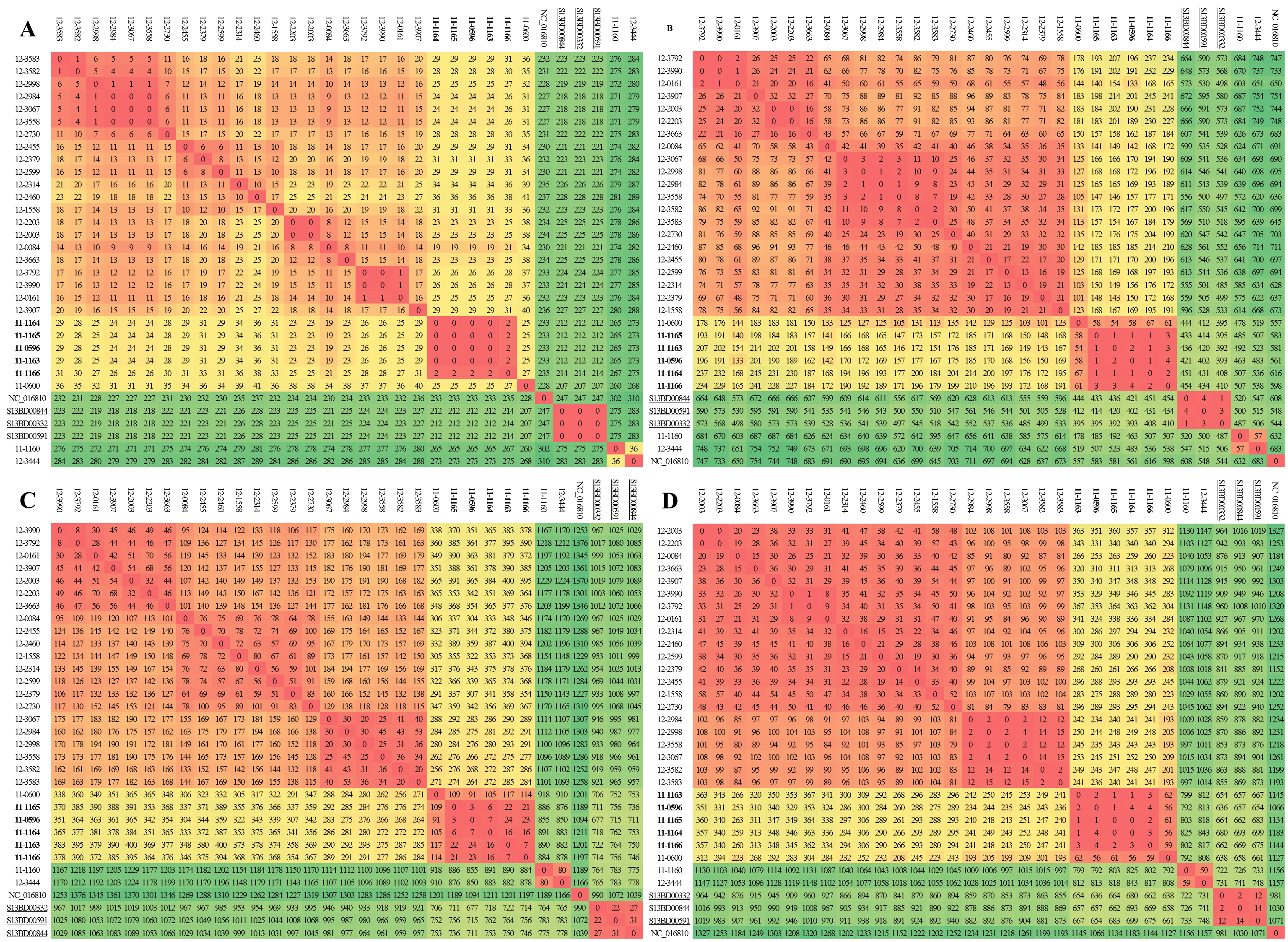

Supplement: S14 Fig — (A) CSI-based workflow, (B) PHEnix-based workflow, (C) CFSAN-based workflow, (D) adapted CFSAN-based workflow. Values and colour codes in the SNP distance matrices indicate pairwise SNP distances between isolates. Outbreak isolates are shown in bold and isolates obtained from the same patient are underlined. (TIF) [file pone.0192504.s014.tif]

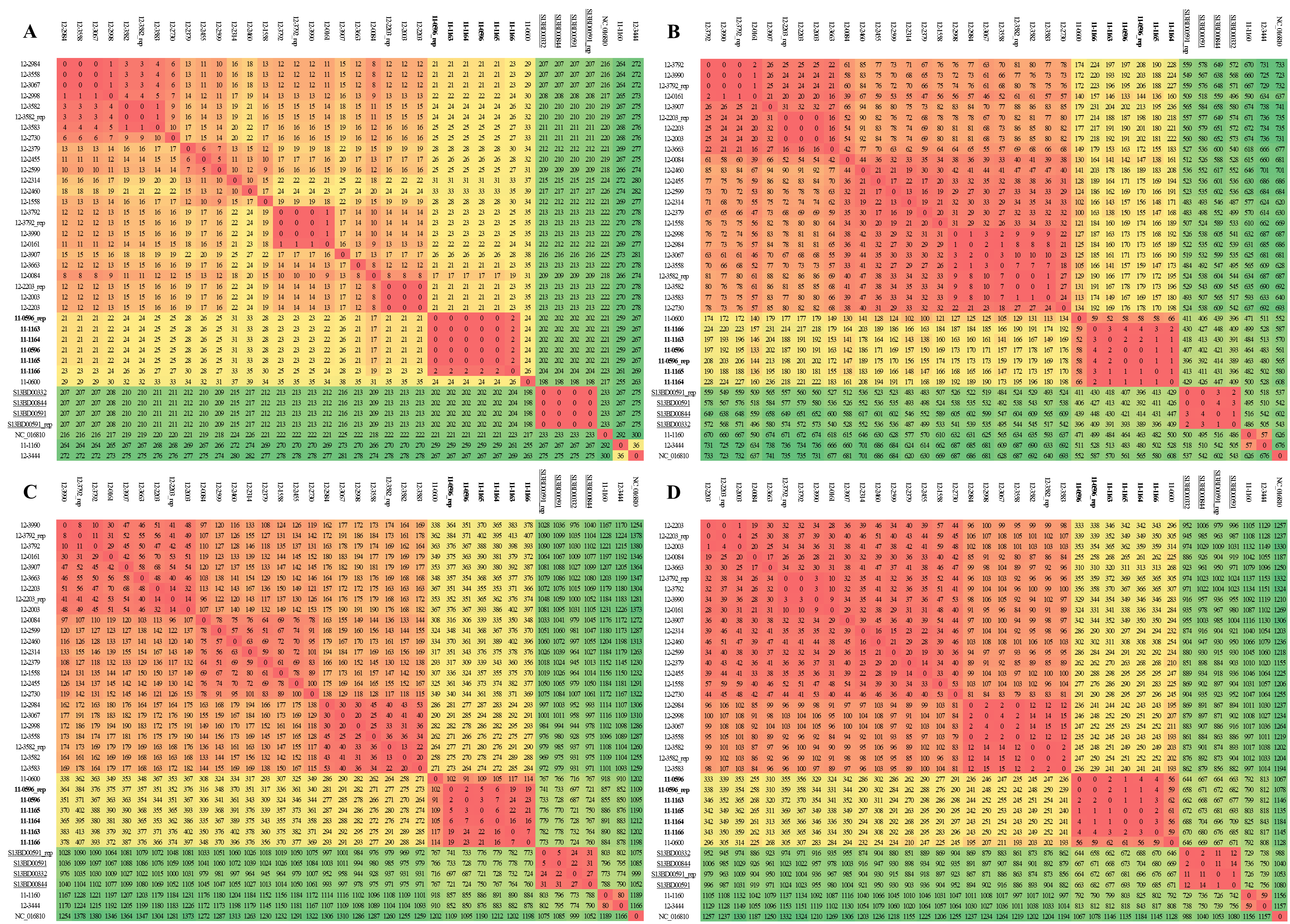

Supplement: S15 Fig — (A) CSI-based workflow, (B) PHEnix-based workflow, (C) CFSAN-based workflow, (D) adapted CFSAN-based workflow. Values and colour codes in the SNP distance matrices indicate pairwise SNP distances between isolates. Outbreak isolates are shown in bold and isolates obtained from the same patient are underlined. (TIF) [file pone.0192504.s015.tif]
