# Supplementary material for: Comparison of SNP-based subtyping workflows for bacterial isolates using WGS data, applied to Salmonella enterica serotype Typhimurium and serotype 1,4,[5],12:i:-
Source: PLoS One. 2018 Feb 6;13(2):e0192504. doi: 10.1371/journal.pone.0192504 (PMC5800660; doi:10.1371/journal.pone.0192504)
Supplement: S1 Table — Performance metrics of the workflows were measured using original dataset (OD) and dataset down-sampled to a 30X coverage (30X), with LT2 as a reference genome. ad. CFSAN-based workflow: adapted CFSAN-based workflow. PHEnix + CSI, PHEnix + CFSAN, etc.: refer to a combination of the variant calling rules from the first mentioned workflow with the SNP matrix construction rules of the second mentioned workflow. DP: discriminative power. (DOCX) [file pone.0192504.s016.docx]

**S1 Table. Performance metrics describing the output of tested SNP-based subtyping workflows and combinations thereof assessed using LT2 as a reference genome.**

|  | **CSI-based workflow** | | **PHEnix-based workflow** | | **ad. PHEnix-based workflow** | | **CFSAN-based workflow** | | **ad. CFSAN-based workflow** | | **PHEnix + CSI** | | **PHEnix + CFSAN** | | **CFSAN + CSI** | | **CFSAN + PHEnix** | |
| --- | --- | --- | --- | --- | --- | --- | --- | --- | --- | --- | --- | --- | --- | --- | --- | --- | --- | --- |
|  | **OD** | **30X** | **OD** | **30X** | **OD** | **30X** | **OD** | **30X** | **OD** | **30X** | **OD** | **30X** | **OD** | **30X** | **OD** | **30X** | **OD** | **30X** |
| *Epidemiologic concordance* | 1 | 1 | 1 | 1 | 0.6 | 0.6 | 1 | 1 | 1 | 1 | 1 | 1 | 1 | 1 | 1 | 1 | 1 | 1 |
| *SNP matrix size* | 999 | 537 | 1649 | 1056 | 2089 | 1880 | 2008 | 1732 | 2226 | 1830 | 1242 | 681 | 2089 | 1880 | 1704 | 1345 | 1874 | 1661 |
| *Number of subtypes* | 28 | 23 | 29 | 28 | 32 | 32 | 32 | 32 | 30 | 30 | 28 | 24 | 30 | 29 | 30 | 31 | 32 | 32 |
| *DP* | 0.992 | 0.972 | 0.994 | 0.990 | 1.00 | 1.00 | 1.00 | 1.00 | 0.996 | 0.996 | 0.992 | 0.976 | 0.996 | 0.994 | 0.996 | 0.998 | 1.00 | 1.00 |
| *Confidence interval of DP* | 0.982-1.00 | 0.947-0.996 | 0.985-1.00 | 0.976-1.00 | 1.00-1.00 | 1.00-1.00 | 1.00-1.00 | 1.00-1.00 | 0.989-1.00 | 0.989-1.00 | 0.982-1.00 | 0.952-0.999 | 0.989-1.00 | 0.985-1.00 | 0.989-1.00 | 0.993-1.00 | 1.00-1.00 | 1.00-1.00 |

Performance metrics of the workflows were assessed using original dataset (OD) and dataset downsampled to a 30X coverage (30X), with LT2 as a reference genome. ad. CFSAN-based workflow: adapted CFSAN-based workflow. PHEnix + CSI, PHEnix + CFSAN , etc.: refer to a combination of the variant calling rules from the first mentioned workflow with the SNP matrix construction rules of the second mentioned workflow. DP: discriminative power.
