# Supplementary material for: Comparison of SNP-based subtyping workflows for bacterial isolates using WGS data, applied to Salmonella enterica serotype Typhimurium and serotype 1,4,[5],12:i:-
Source: PLoS One. 2018 Feb 6;13(2):e0192504. doi: 10.1371/journal.pone.0192504 (PMC5800660; doi:10.1371/journal.pone.0192504)
Supplement: S2 Table — Performance metrics of the workflows were measured using original dataset (OD) and dataset down-sampled to a 30X coverage (30X), with SL1344 as a reference genome. ad. CFSAN-based workflow: adapted CFSAN-based workflow. PHEnix + CSI, PHEnix + CFSAN, etc.: refer to a combination of the variant calling rules from the first mentioned workflow with the SNP matrix construction rules of the second mentioned workflow. DP: discriminative power. (DOCX) [file pone.0192504.s017.docx]

**S2 Table. Performance metrics describing the output of tested SNP-based subtyping workflows and combinations thereof assessed using SL1344 as a reference genome.**

|  | **CSI-based workflow** | | **PHEnix-based workflow** | | **ad. PHEnix-based workflow** | | **CFSAN-based workflow** | | **ad. CFSAN-based workflow** | | **PHEnix + CSI** | | **PHEnix + CFSAN** | | **CFSAN + CSI** | | **CFSAN + PHEnix** | |
| --- | --- | --- | --- | --- | --- | --- | --- | --- | --- | --- | --- | --- | --- | --- | --- | --- | --- | --- |
|  | **OD** | **30X** | **OD** | **30X** | **OD** | **30X** | **OD** | **30X** | **OD** | **30X** | **OD** | **30X** | **OD** | **30X** | **OD** | **30X** | **OD** | **30X** |
| *SNP matrix size* | 1239 | 629 | 2693 | 1554 | 3431 | 3058 | 2982 | 2519 | 3273 | 2630 | 1740 | 823 | 3431 | 3058 | 2372 | 1836 | 2755 | 2389 |
| *Number of subtypes* | 28 | 23 | 30 | 30 | 32 | 32 | 32 | 32 | 31 | 29 | 29 | 24 | 32 | 31 | 32 | 32 | 32 | 32 |
| *DP* | 0.992 | 0.972 | 0.996 | 0.996 | 1.00 | 1.00 | 1.00 | 1.00 | 0.998 | 0.994 | 0.994 | 0.976 | 1 | 0.998 | 1 | 1 | 1.00 | 1.00 |
| *Confidence interval of DP* | 0.982-1.00 | 0.947-0.996 | 0.989-1.00 | 0.989-1.00 | 1.00-1.00 | 1.00-1.00 | 1.00-1.00 | 1.00-1.00 | 0.993-1.00 | 0.985-1.00 | 0.985-1.00 | 0.952-0.999 | 1.00-1.00 | 0.993-1.00 | 1.00-1.00 | 1.00-1.00 | 1.00-1.00 | 1.00-1.00 |

Performance metrics of the workflows were obtained using original dataset (OD) and dataset downsampled to a 30X coverage (30X), with SL1344 as a reference genome. ad. CFSAN-based workflow: adapted CFSAN-based workflow. PHEnix + CSI, PHEnix + CFSAN , etc.: refer to a combination of the variant calling rules from the first mentioned workflow with the SNP matrix construction rules of the second mentioned workflow. DP: discriminative power.
