# Supplementary material for: Comparison of SNP-based subtyping workflows for bacterial isolates using WGS data, applied to Salmonella enterica serotype Typhimurium and serotype 1,4,[5],12:i:-
Source: PLoS One. 2018 Feb 6;13(2):e0192504. doi: 10.1371/journal.pone.0192504 (PMC5800660; doi:10.1371/journal.pone.0192504)
Supplement: S1 File — (DOCX) [file pone.0192504.s018.docx]

#!/usr/bin/perl

use strict;

use warnings;

# Usage ./sampleFastq.pl <fastq r1> <fastq r2> <outFastq r1> <outFastq r2> <prob

of keeping reads>

# ./sampleFastq.pl <(gunzip -c f.fastq.gz) <(gunzip -c r.fastq.gz) >(gzip -c - >

f_sample.fastq.gz) >(gzip -c - > r_sample.fastq.gz) 0.5

open(FASTQF,$ARGV[0]);

open(FASTQR,$ARGV[1]);

open(FASTQOUTF,">".$ARGV[2]);

open(FASTQOUTR,">".$ARGV[3]);

my $proba = $ARGV[4];

my $line1;

my $line2;

my $nbLines = 1;

my $random;

my $fqRecord1;

my $fqRecord2;

while($line1=<FASTQF>){

$line2=<FASTQR>;

$fqRecord1.=$line1;

$fqRecord2.=$line2;

if($nbLines%4==0){

$random = rand(1);

if($random <= $proba){

print FASTQOUTF $fqRecord1;

print FASTQOUTR $fqRecord2;

}

$fqRecord1="";

$fqRecord2="";

}

$nbLines++;

}

close(FASTQOUTR);

close(FASTQOUTF);

close(FASTQR);

close(FASTQF);
